# Seroconversion and fever are dose-dependent in a nonhuman primate model of inhalational COVID-19

**DOI:** 10.1371/journal.ppat.1009865

**Published:** 2021-08-23

**Authors:** Paul A. Dabisch, Jennifer Biryukov, Katie Beck, Jeremy A. Boydston, Jaleal S. Sanjak, Artemas Herzog, Brian Green, Gregory Williams, John Yeager, Jordan K. Bohannon, Brian Holland, David Miller, Amy L. Reese, Denise Freeburger, Susan Miller, Tammy Jenkins, Sherry Rippeon, James Miller, David Clarke, Emmanuel Manan, Ashley Patty, Kim Rhodes, Tina Sweeney, Michael Winpigler, Owen Price, Jason Rodriguez, Louis A. Altamura, Heather Zimmerman, Alec S. Hail, Victoria Wahl, Michael Hevey

**Affiliations:** 1 National Biodefense Analysis and Countermeasures Center (NBACC), Operated by Battelle National Biodefense Institute for the US Department of Homeland Security, Frederick, Maryland, United States of America; 2 Gryphon Scientific LLC, Takoma Park, Maryland, United States of America; 3 Censeo Insight, Seattle, Washington, United States of America; 4 Applied Research Associates, Arlington, Virginia, United States of America; Johns Hopkins University Bloomberg School of Public Health, UNITED STATES

## Abstract

While evidence exists supporting the potential for aerosol transmission of SARS-CoV-2, the infectious dose by inhalation remains unknown. In the present study, the probability of infection following inhalation of SARS-CoV-2 was dose-dependent in a nonhuman primate model of inhalational COVID-19. The median infectious dose, assessed by seroconversion, was 52 TCID_50_ (95% CI: 23–363 TCID_50_), and was significantly lower than the median dose for fever (256 TCID_50_, 95% CI: 102–603 TCID_50_), resulting in a group of animals that developed an immune response post-exposure but did not develop fever or other clinical signs of infection. In a subset of these animals, virus was detected in nasopharyngeal and/or oropharyngeal swabs, suggesting that infected animals without signs of disease are able to shed virus and may be infectious, which is consistent with reports of asymptomatic spread in human cases of COVID-19. These results suggest that differences in exposure dose may be a factor influencing disease presentation in humans, and reinforce the importance of public health measures that limit exposure dose, such as social distancing, masking, and increased ventilation. The dose-response data provided by this study are important to inform disease transmission and hazard modeling, and, ultimately, mitigation strategies. Additionally, these data will be useful to inform dose selection in future studies examining the efficacy of therapeutics and vaccines against inhalational COVID-19, and as a baseline in healthy, young adult animals for assessment of the importance of other factors, such as age, comorbidities, and viral variant, on the infectious dose and disease presentation.

## Author summary

SARS-CoV-2 infection and subsequent disease presentation are dependent on exposure dose in a nonhuman primate model of inhalational COVID-19.

## Introduction

Exhalation of aerosols containing infectious SARS-CoV-2 represents a potential mode of transmission for COVID-19. In order to understand the hazard posed by aerosols containing SARS-CoV-2, information on a number of parameters are needed, including the emission rate and size distribution of particles from an infected individual, the ability of infectious virus contained within those particles to survive in the air, and the infectivity following inhalation by a susceptible host. Previous studies have demonstrated that significant quantities of aerosol particles can be generated from the respiratory tract during different activities, including breathing, speaking, singing, and coughing [1–4]. Multiple studies have also demonstrated that aerosol particles exhaled by individuals with respiratory infections, including COVID-19, can contain infectious virus or viral RNA [5–11]. Air sampling has detected infectious SARS-CoV-2 or viral RNA in clinical settings [5, 6,12–14], and surface sampling in the same settings has detected viral RNA on room exhaust vents, again suggesting the presence of aerosol particles containing SARS-CoV-2 [12,15]. A significant fraction of the measured airborne particles detected in these studies had aerodynamic diameters less than 5 μm [1–4,9,12,14]. Such particles are potentially hazardous as they would remain airborne for extended periods of time, and multiple studies have demonstrated that SARS-CoV-2 present in aerosol particles in this size range can remain infectious for long durations under some conditions [16–21]. Finally, it has been shown that nonhuman primates exposed to aerosols with aerodynamic diameters less than 5 μm containing SARS-CoV-2 develop disease similar to COVID-19 in humans [22–26]. Taken together, these studies suggest that transmission of SARS-CoV-2 by aerosols generated from the respiratory tract may be possible. However, significant knowledge gaps remain, including the magnitude and time course of shedding of infectious viral aerosols from infected individuals, host factors that may influence generation of respiratory aerosols, and the dose-infectivity relationship for SARS-CoV-2 by inhalation.

Numerous studies have examined disease presentation in animal models of COVID-19 [27]. The majority of these studies induce infection via either intratracheal or intranasal instillation of a bolus of a viral suspension [22,28–30]. However, while direct instillation into the lungs via the trachea or nasal cavity has been employed in past studies as an alternative to inhalation exposure, it should be recognized that the distribution of the inoculum throughout the respiratory tract differs significantly between these routes of administration [31]. Previous studies with other microorganisms have demonstrated that differences in the regional deposition in the respiratory tract can significantly alter the lethal dose of an inhaled pathogen, as well as the disease presentation and time course [32–35]. In several studies with SARS-CoV-2, a similar disease presentation was observed in nonhuman primates following exposure to either aerosolized virus or a multi-route bolus mucosal exposure paradigm despite an orders of magnitude difference in the doses reported for the two exposure paradigms [23,25]. However, the difference in the regional deposition fractions within respiratory and gastrointestinal tracts between the two routes of exposure is not clear, making it difficult to discern the role of regional deposition in animal models of COVID-19 without further study. These studies highlight the potential importance of route of exposure in the development of animal models of disease, and the need for studies assessing the influence of route of exposure on disease presentation in animal models of COVID-19.

To date, only a few studies have examined disease presentation following inhalation of aerosolized SARS-CoV-2, despite the potential relevance of this route of exposure [22–26]. Furthermore, these studies only examined disease presentation following exposure to a single dose of SARS-CoV-2. Thus, while dose-infectivity data are needed to inform disease transmission and hazard modeling, and, ultimately, inform mitigation strategies, no such data currently exist for SARS-CoV-2. Therefore, the aim of the present study was to examine the relationship between exposure dose and disease presentation in a nonhuman primate model of inhalational COVID-19. Exposures utilized small particle aerosols containing SARS-CoV-2, with aerodynamic diameters less than 5 μm, in order to mimic the expected particle size distributions generated during breathing or speaking. Both seroconversion and fever were dose-dependent, with seroconversion occurring at significantly lower doses than fever. The dose-response models generated by this study provide insight into the influence of exposure dose on disease presentation and may help to explain the range of different disease presentations observed in the human population. Finally, the animal model developed as part of this study will provide a baseline in healthy young adult animals against which it will be possible to compare and assess the importance of other factors associated with more severe disease in humans, such as age, comorbidities, viral variant, and route of exposure.

## Results

Sixteen healthy, young adult cynomolgus macaques (*Macaca fascicularis*; 8 male and 8 female) were exposed to small particle aerosols containing SARS-CoV-2, with calculated deposited doses ranging from approximately 5 to 906 TCID_50_. The average mass median aerodynamic diameter (MMAD) and geometric standard deviation (GSD) for the generated aerosols were 1.44±0.10 μm and 1.54±0.04, respectively (n = 16). A summary of exposure parameters and doses for all animals is provided in Table A in S1 Text.

**Fig 1:**
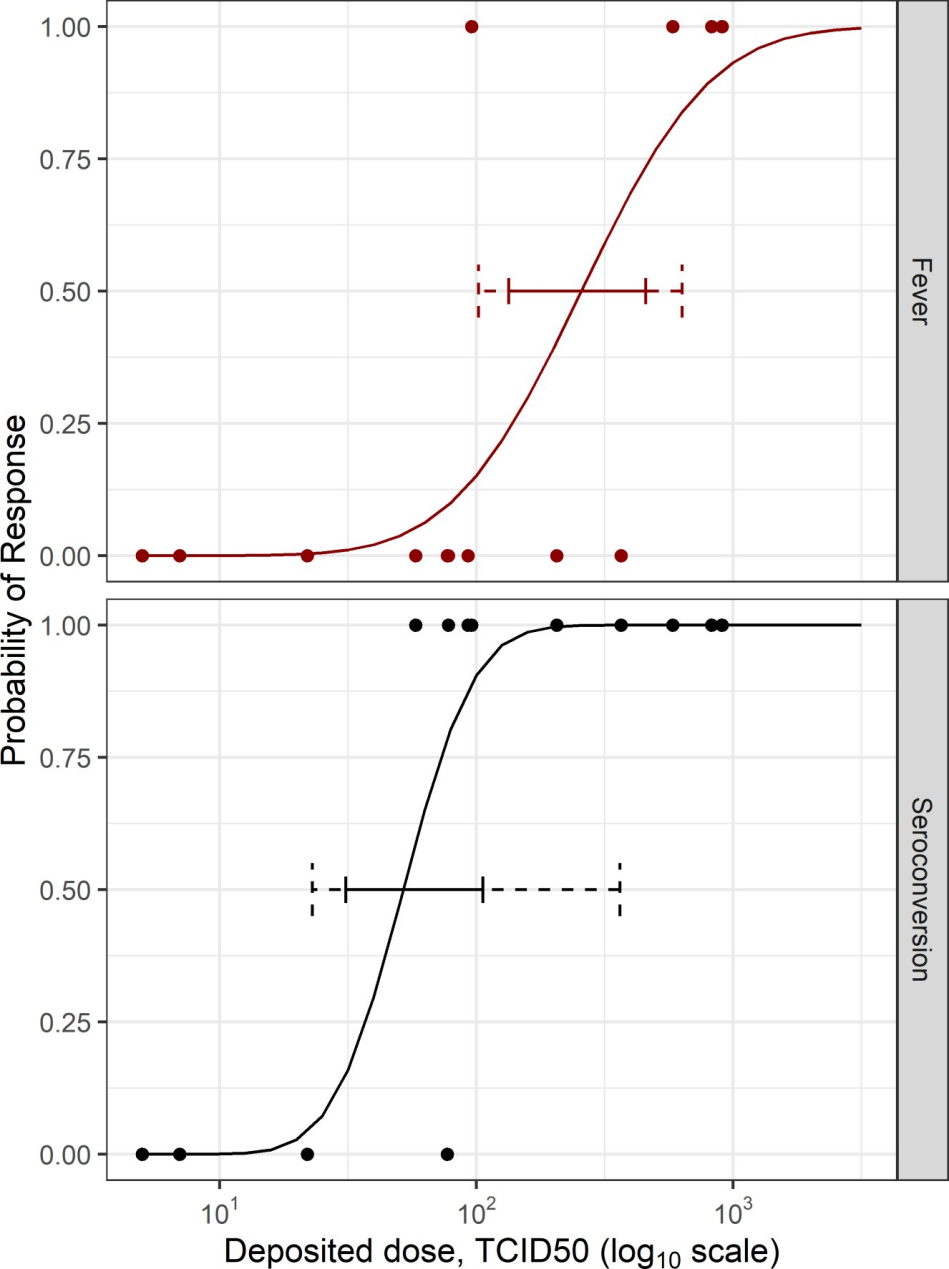
Dose-response relationships for fever and seroconversion. The dashed and solid horizontal line segments indicate the 95% and 85% bias-corrected and accelerated bootstrap percentile confidence intervals, respectively. For fever, the estimated median dose was 256 TCID_50_, with 95% and 85% confidence intervals of 102–633 TCID_50_ and 134–458 TCID_50_, respectively. For seroconversion, the estimated median dose was 52 TCID_50_, with estimated 95% and 85% confidence intervals of 23–363 TCID_50_ and 31–106 TCID_50_, respectively. The median deposited dose for seroconversion was significantly lower than that estimated for fever when compared using the LRT (P = 0.03).

Ten of 16 animals were infected by the end of the post-exposure observation period on day 21, as assessed by seroconversion ELISA and quantification of neutralizing antibody titers. Seroconversion was dose-dependent, with the median deposited dose for seroconversion estimated to be 52 TCID_50_ (95% CI: 23–363 TCID_50_; 85% CI: 31–106 TCID_50_; Fig 1). In animals that seroconverted, a plaque reduction neutralization test (PRNT) was conducted to estimate neutralizing titers, if present. Neutralizing antibodies were present in all animals that seroconverted, with peak PRNT_50_ titers ranging from 80 to 640 (Table 1). Peak neutralizing titers did not correlate with dose (R^2^<0.001 using linear regression; slope not significantly different than zero). Additionally, there was not a strong relationship between deposited dose and time to seroconversion (R^2^ = 0.01 using linear regression; slope not significantly different than zero), although the frequency of blood draws decreased from every other day to weekly after day 10 post-exposure, complicating assessment of this relationship. Complete time course data for seroconversion and PRNT titers for each animal are provided in Tables B and C in S1 Text.

**Table 1:** Summary of fever and seroconversion results by animal.

Animal	Deposited Dose		Fever (Yes/No)	Time to Fever (HPE)	Seroconversion (Yes/No)	Time to Seroconversion (DPE)	Peak PNRT_50_ titer
	TCID_50_	log_10_ TCID_50_					
1	5	0.73	No		No		
2	7	0.82	No		No		
3	7	0.86	No		No		
4	17	1.23	ND		No		
5	22	1.35	No		No		
6	58	1.76	No		Yes	14	160
7	77	1.88	No		No		
8	78	1.89	No		Yes	14	640
9	93	1.97	No		Yes	21	80
10	96	1.98	Yes	36	Yes	14	640
11	206	2.31	No		Yes	14	160
12	366	2.56	No		Yes	21	640
13	582	2.77	Yes	17.75	Yes	14	320
14	825	2.92	Yes	41.25	Yes	21	80
15	904	2.96	Yes	30.25	Yes	10	640
16	906	2.96	Yes	34	Yes	14	160

HPE: Hours post-exposure; DPE: Days post-exposure; ND: no data due to malfunctioning telemetry device; Grayed out samples = not applicable.

**Fig 2:**
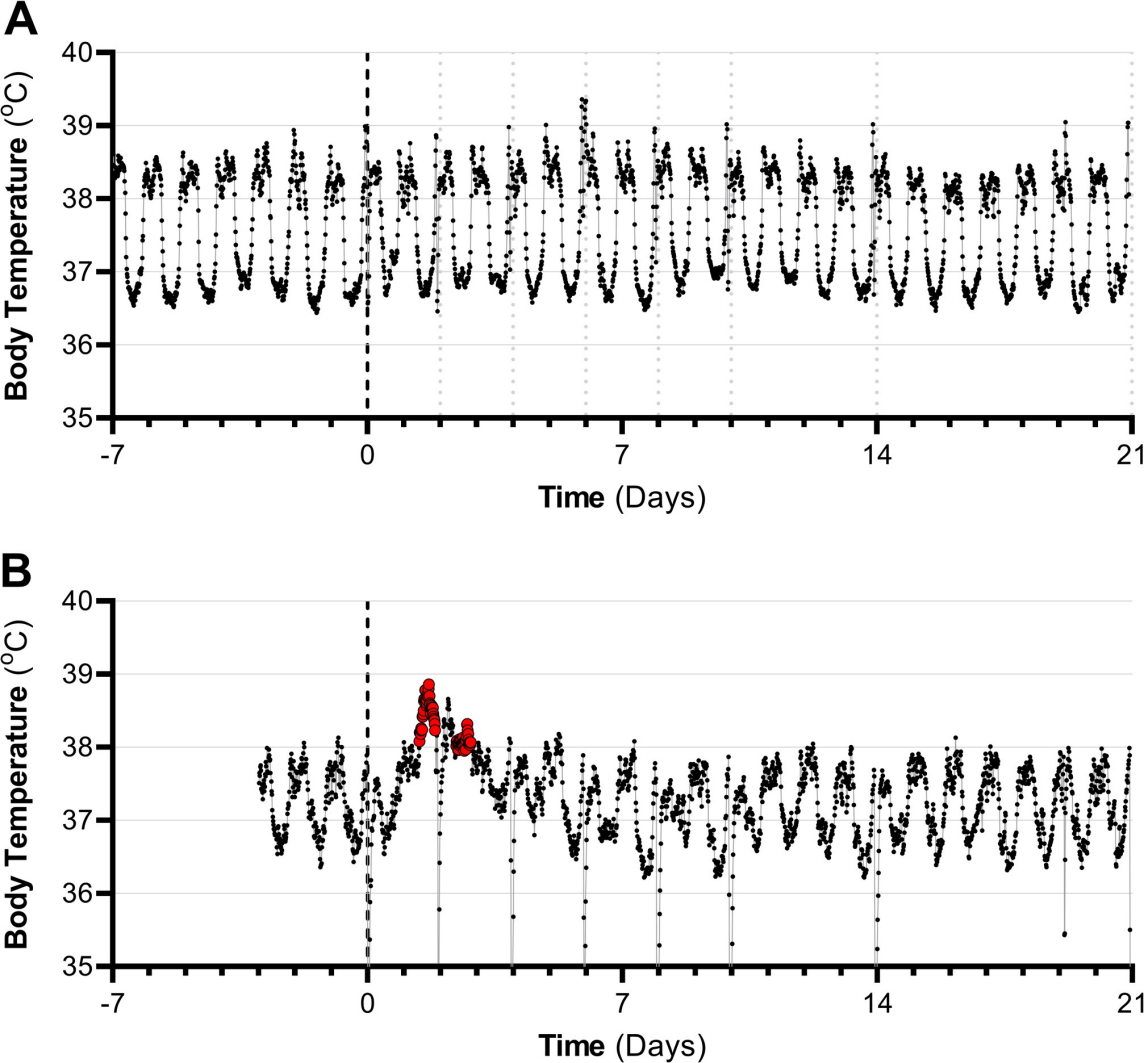
Representative temperature profiles. (A) Temperature profile for animal that received a deposited dose of 58 TCID_50_ and did not develop fever, but seroconverted on day 14 post-exposure; (B) Temperature profile for animal which received a deposited dose of 906 TCID_50_ that developed fever and seroconverted on day 14 post-exposure. Red circles represent periods of fever in which the body temperature was >1°C above baseline for more than two hours. Dark vertical dashed line at day 0 represents exposure. Lighter vertical lines at days 2, 4, 6, 8, 10, 14, and 21 indicate administration of anesthesia for collection of blood and swab samples. Decreases in body temperature are evident upon administration of anesthesia, and disrupt the period of continuous fever observed in the animal shown in (B).

Transient periods of fever, defined as an increase in body temperature of >1°C above baseline for greater than 2 hours, were observed in five of 16 animals. The presence of fever was also dose-dependent, with the median deposited dose for fever estimated to be 256 TCID_50_ (95% CI: 102–603 TCID_50_; 85% CI: 134–458 TCID_50_; Fig 1). The median time to onset of fever was 34 hours, with a range of 17.75 to 41.25 hours. Representative temperature profiles for two animals, both of which seroconverted but only one of which developed a fever, are shown in Fig 2. While seroconversion occurred in all animals with fever, it occurred multiple days after the onset of fever (Table 1). The longest duration of continuous fever observed in each animal ranged from 2.5 to 12.75 hours. A single continuous period of fever was observed in one animal, while multiple separate periods of fever were observed in the other four animals. There was not a strong relationship between deposited dose and either the time to fever onset, the longest duration of fever, or the number of fever periods (R^2^ = 0.14, 0.04, and 0.19, respectively, using linear regression; slopes not significantly different than zero). However, these analyses should be interpreted with caution given the limited number of animals with fever and the relative proximity of the deposited doses for these animals.

Fever and seroconversion are likely to be physiologically related, and the data in Table 1 show marginal evidence for correlation between the two endpoints (P = 0.08 by Fisher’s exact test). Since fever and seroconversion data are paired by subject and given the potential for correlation, the two endpoints were modeled jointly in a bivariate vector generalized linear model [36,37]. A bivariate model where fever and seroconversion were allowed to have different overall dose response parameters was a better fit to the data than a similar model wherein the dose response parameters for the two endpoints were constrained to be equivalent (P = 0.02). The median deposited dose for seroconversion was significantly lower than that estimated for fever when compared using the likelihood ratio test (LRT; P = 0.03). Additionally, the 85% confidence intervals for the median responses, which have been shown to be a reliable indicator of significance for independent dose response models, do not overlap [38] (Fig 1). A model where fever is dose dependent when conditioned on seroconversion was a better fit to the data than a model with a constant probability of fever conditional upon seroconversion (P = 0.02). Taken together, these results suggest that higher doses are needed to induce seroconversion with fever as opposed to seroconversion alone. Additionally, the dose-response models predict that fever alone would be a rare occurrence.

For comparison of viral shedding, cytokine levels, hematology, serum chemistry, and clotting parameters, animals were grouped by disease presentation, specifically those animals that had fever and seroconverted (n = 5), those that seroconverted but did not have fever (n = 5), and those animals that neither seroconverted nor had fever (n = 6). Oropharyngeal and nasopharyngeal swabs were collected throughout the post-exposure period and analyzed for the presence of infectious virus and viral RNA. Intermittent shedding of either infectious virus or viral RNA was detected in eight of the 10 animals that seroconverted, and in all five animals that developed fever. Low levels of viral RNA were also detected on single days in two of the six animals that did not seroconvert, and a low level of infectious virus was also recovered in one of these animals on a single day (day 6 post-exposure). The proportion of all post-exposure samples positive for infectious virus or viral RNA was similar in the group of infected animals with fever (33/140 samples) and the group of infected animals without fever (24/140 samples; P = 0.23 by Fisher’s exact test). However, both were significantly greater than the proportion observed in the group without seroconversion/fever (4/168 samples; P<0.0001 by Fisher’s exact tests). Levels of infectious virus recovered from swabs ranged from 0.6 to 3.3 log_10_ TCID_50_/mL, and were detected as early as two days post-exposure and as late ten days post-exposure. Levels of viral RNA recovered from swabs ranged from 4.3 to 8.7 log_10_ RNA copies/mL, and were detected as early as two days post-exposure and as late as day 21 post-exposure. A total of 16 samples were positive by both PCR and microtitration. The ratio of RNA copies to TCID_50_ was not significantly different between oropharyngeal and nasopharyngeal swabs (P = 0.62), with a mean ± standard deviation of 5.6 ± 0.7 log_10_ RNA copies per TCID_50_ (n = 16) pooled across swab types. A summary of peak levels of viral shedding by disease presentation is shown in Tables 2 and 3, and complete time course data for viral shedding for each swab type, for each animal, are provided in Tables D-G in S1 Text.

**Table 2:** Summary of infectious viral shedding in oropharyngeal and nasopharyngeal swabs.

Disease Presentation	Nasopharyngeal Swabs			Oropharyngeal Swabs		
	Median Peak log_10_ TCID_50_/mL (Range)	Median time (days) to peak (Range)	n	Median Peak log_10_ TCID_50_/mL (Range)	Median time (days) to peak (Range)	n
Seroconversion and Fever (n = 5)	2.8 (0.6–3.3)	6 (2–8)	3	0.8 (0.6–1.1)	5 (2–8)	4
Seroconversion, No fever (n = 5)	2.1 (1.0–3.2)	5 (4–6)	2	0.8 (NA)	2 (NA)	1
No seroconversion, No fever (n = 6)	NA (NA)	NA (NA)	0	0.8 (NA)	6(NA)	1

NA: not applicable.

**Table 3:** Summary of viral RNA shedding in oropharyngeal and nasopharyngeal swabs.

Disease Presentation	Nasopharyngeal Swabs			Oropharyngeal Swabs		
	Median Peak log_10_ RNA copies/mL (Range)	Median time (days) to peak (Range)	n	Median Peak log_10_ RNA copies/mL (Range)	Median time (days) to peak (Range)	n
Seroconversion and Fever (n = 5)	6.1 (4.6–8.7)	2 (2–8)	5	6.3 (5.4–6.8)	5 (2–8)	4
Seroconversion, No fever (n = 5)	8.3 (8.1–8.5)	5 (4–6)	2	5.5 (4.6–6.4)	2 (2)	3
No seroconversion, No fever (n = 6)	5.4 (NA)	21 (NA)	1	5.8 (4.9–6.7)	4 (2–6)	2

NA: not applicable.

**Fig 3:**
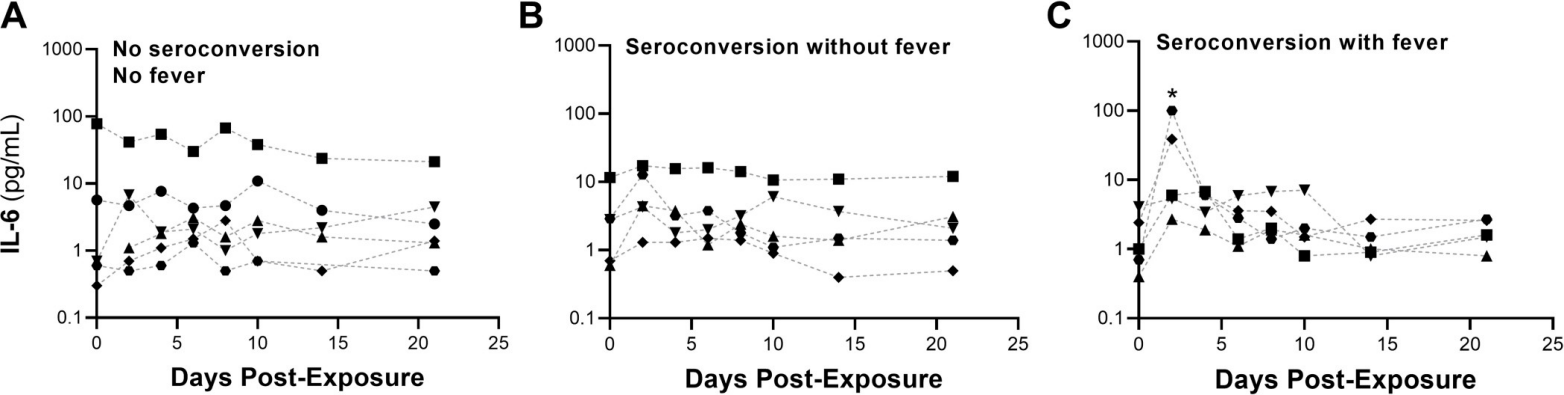
Serum IL-6 Profiles. (A) IL-6 serum concentrations over time are shown for animals that did not seroconvert or have fever (n = 6). IL-6 concentrations at all post-exposure time points were not significantly different than the baseline IL-6 levels. One animal in this group had a significantly higher baseline and post-exposure levels than the other animals, and appears to be an outlier. (B) IL-6 serum concentrations over time are shown for animals that seroconverted but did not have fever (n = 5). IL-6 concentrations at all post-exposure time points were not significantly different than the baseline IL-6 levels. (C) IL-6 serum concentrations over time are shown for animals that seroconverted and had fever (n = 5). IL-6 levels increased on day 2 in all animals in this group relative to baseline, but two of the five animals had increases that were much greater than the others (>35 pg/mL, and >10x baseline), reaching levels reported for severe cases of COVID-19 in humans. IL-6 levels returned to baseline levels by day 4 post-exposure; * denotes P<0.05 when compared to the day 0 baseline levels.

In the group with both seroconversion and fever, IL-6 levels post-exposure were transiently, but significantly, increased on day 2 relative to baseline (baseline range: 0.4–4.1 pg/mL; day 2 post-exposure range: 2.7–100.5 pg/mL; P = 0.035; Fig 3). IL-6 levels were increased on day 2 in all animals in this group relative to baseline, but two of the five animals had increases that were much greater than the others (>35 pg/mL and >10x over baseline), reaching levels reported for severe cases of COVID-19 in humans [39,40]. IL-6 levels returned to baseline levels by day 4 post-exposure (P = 0.06). All other post-exposure time points in all three groups were not significantly different than the baseline IL-6 levels (P>0.06). Similar analyses performed for serum levels of IL-8 showed that there were not any significant changes from baseline over the course of the post-exposure period (P>0.16). Complete time course data for IL-6 and IL-8 are provided in Tables H and I in S1 Text. Similar analyses were not performed for other cytokines, specifically IL-10, IL-2, IL-1β, and IFN-γ, as the levels detected in the majority of samples were below the limit of detection of the assay.

Increases in the neutrophil:lymphocyte ratio (NLR) have been reported in patients with more severe COVID-19 [41,42]. In the present study, no significant changes from baseline were observed for the NLR (P>0.20) in any of the groups at any post-exposure time point. Complete time course data for the NLR are provided in Table J in S1 Text.

Coagulation abnormalities have also been reported in patients with COVID-19, including increased D-dimer levels, prolonged prothrombin times (PT), prolonged activated partial thromboplastin times (aPTT), and mild thrombocytopenia [43,44]. In the present study, no significant increases from baseline were observed for either the PT (P>0.12) or aPTT (P>0.19) in any group at any time point post-exposure. However, a small but significant decrease in the PT (P<0.02; mean decrease of 1.0–1.1 sec) was observed at several post exposure time points in several of the groups. Similarly, a small but significant decrease from baseline in the aPTT (P<0.01; mean decrease of 2.7 sec) was observed at 4 days post-exposure in the group without seroconversion or fever. Complete time course data for the PT and aPTT are provided in Tables K and L in S1 Text.

Changes in kidney function have been associated with more severe disease in human cases of COVID-19, specifically, increases in blood urea nitrogen (BUN), creatinine (CRE), and the BUN:CRE ratio [45–47]. In the present study, no significant increases from baseline were observed for BUN levels (P> 0.10), creatinine (CRE) levels (P> 0.12), or the BUN:CRE ratio (P> 0.10) in any of the groups at any time point post-exposure. However, small but significant decreases (P<0.05) relative to baseline were observed for BUN and BUN:CRE ratio at several of the time points post-exposure. Complete time course data for BUN, CRE, and BUN:CRE are provided in Tables M-O in S1 Text.

For comparison of body weights, animals were grouped similarly to analyses performed for comparison of cytokine levels. No significant changes in body weight were observed during the first 10 days post-exposure in any of the groups when compared using the non-parametric Friedman test (P>0.19 for all comparisons). Complete time course data for body weight are provided in Table P in S1 Text. Additionally, no respiratory signs, or changes in activity were noted during twice-daily observations.

## Discussion

The present study is the first to demonstrate that the probability of infection is dependent on the exposure dose of SARS-CoV-2 in a nonhuman primate model of inhalational COVID-19. The results demonstrate that the probability of seroconversion and fever are both dose-dependent, but that the median dose for seroconversion is significantly lower than that of fever, resulting in a group of animals that developed an immune response post-exposure but did not develop fever or other clinical signs of infection. In a subset of animals in this group, virus was detected in nasopharyngeal and/or oropharyngeal swabs, suggesting that infected animals without signs of disease are shedding virus and may be infectious, which is consistent with reports of asymptomatic spreaders in human cases of COVID-19 [48]. These results suggest that differences in exposure dose may be a factor influencing disease presentation in humans, as has been suggested by several epidemiological investigations [49,50], and reinforce the importance of public health measures that limit exposure dose, such as social distancing, masking, and increased ventilation. The dose-response data provided by this study are important to inform disease transmission and hazard modeling, and, ultimately, inform mitigation strategies. Additionally, these data will be useful to inform dose selection in future studies examining the efficacy of therapeutics and vaccines against inhalational COVID-19, and as a baseline for assessment of the importance of other factors, such as age, comorbidities, and viral variant, on the infectious dose and disease presentation.

The healthy young adult cynomolgus macaques utilized in the present study developed mild, dose-dependent disease following inhalational exposure to SARS-CoV-2. At higher doses, infection was characterized by transient fever with an onset within 48 hours of exposure, seroconversion and development of neutralizing antibody titers between 10 and 21 days following exposure, and intermittent shedding of virus in nasopharyngeal and/or oropharyngeal swabs. No respiratory signs, changes in body weight, or changes in activity were observed. These results are consistent with reports of milder disease in healthy young adult humans [51,52], and suggest that the cynomolgus macaque may be a reasonable model of mild disease. These results are also consistent with those of several previous studies that examined disease presentation in nonhuman primate models of inhalational COVID-19, which utilized inhaled doses of SARS-CoV-2 that were greater than those utilized in the present study [25,26]. Hartman et al. [25] reported a mild disease presentation in African green monkeys with transient fever, seroconversion, neutralizing titers, and shedding of replicating virus. Similarly, Johnston et al. [26] reported mild disease with fever and viral shedding in cynomolgus macaques, although the levels of viral shedding reported were greater than those observed in the present study.

Changes in numerous clinical parameters have been shown to be prognostic for development of severe disease in human cases of COVID-19, including NLR, PT, aPTT, BUN, CRE, and BUN:CRE ratio [41–47]. At higher doses when animals had both fever and seroconverted, no significant changes in any of these parameters were observed at any time point during the post-exposure period. These findings are, again, consistent with the observation of mild disease in these animals.

In human cases of COVID-19, elevated levels of IL-6 have been associated with severe disease [40,53]. Similarly, increases in IL-6, in the absence of increases in IL-10, have been associated with disease progression in a nonhuman primate model of COVID-19 [23]. In the present study, a transient, but significant, increase in IL-6 was observed on day 2 post-exposure in animals with fever and seroconversion, including two animals where peak levels were in the range observed in severe human cases. IL-6 levels returned to near baseline levels by 4 to 6 days post-exposure. However, despite the spike in IL-6 levels, disease in these two animals was mild and similar to that of the remaining animals throughout the post-exposure period, suggesting that the duration of the elevation in IL-6 levels may also be important. Despite using higher exposure doses, Hartman et al. [25] did not observe detectable IL-6 at any time point post-exposure, and no other IL-6 time course data were identified in reports on human or animal disease. Thus, additional studies are needed to better elucidate the relationship between the time course of IL-6 concentrations and severity of disease.

In the group of animals that seroconverted and had neutralizing antibody titers, but did not develop fever, no significant changes were observed in the serum or blood parameters already discussed. However, as noted earlier, viral shedding was detected in a subset of these animals by nasopharyngeal and/or oropharyngeal swab samples, suggesting that animals without overt signs of disease may be infectious. As with animals that received higher doses, both infectious virus and viral RNA were intermittently detected throughout the 21-day post-exposure period. These results are similar to those observed in humans, where patients with mild or asymptomatic disease had detectable viral RNA in nasopharyngeal or oropharyngeal swabs for between 6 and 37 days, including several instances of positive results following a previous negative result [54].

Interestingly, transient shedding of viral RNA was also observed in two animals at lower doses that did not develop fever or an antibody response by the end of the study. One of the positive samples occurred on day 2 post-exposure, which could possibly be due to residual RNA from the inhalation exposure, as the RNA concentration detected was low. The other two samples occurred in the same animal, on day 6 and on day 21 post-exposure, and were preceded by negative results. The sample on day 6 was also positive for infectious virus. Thus, it is unlikely that these were due to residual RNA from the exposure. However, it is unclear whether these samples represent localized infection in the upper respiratory tract that did not result in a systemic immune response, or if a low level infection was present that did not result in a systemic immune response of sufficient magnitude to be detected by the end of the post-exposure observation period on day 21.

While the present study provides novel data that will be useful to inform disease transmission and hazard modeling, there are numerous caveats that need to be considered. First, in order to utilize these data in human risk assessment and hazard modeling, appropriate methodologies for extrapolation of dose-response data generated in nonhuman primate models of COVID-19 to humans need to be determined. In the present study, doses are presented as deposited doses, which incorporates a species-specific deposition fraction estimate, normalizing for differences in respiratory geometry and function across species. However, it is unclear if other factors need to be considered for interspecies extrapolation.

Second, the nonhuman primates utilized were serologically naïve, healthy, young adults. Age and co-morbidities are known to affect disease severity in humans [51–53,55], and aged nonhuman primates have been shown to develop more severe disease than younger animals [22,56]. The mechanisms responsible for these differences remain unclear, making it uncertain if the dose-response models developed using healthy, young adult animals are also applicable to other populations. Additional studies to evaluate the impact of age, co-morbidities, as well as the effect of prior exposure to SARS-CoV-2 or vaccination, on the dose response and disease presentation would provide valuable information to further elucidate any differences.

Third, the exposures in the present study utilized small particle aerosols, where the majority of particles had aerodynamic diameters between 1 and 2 μm, meant to mimic the size distribution expected for exhaled particles generated during breathing or speaking. Previous studies have demonstrated that aerosol particle size affects the regional deposition within the respiratory tract, and, subsequently, dose-infectivity relationships and disease presentation for some infectious microorganisms [32,33,35,57,58]. Given that particles with larger aerodynamic diameters would be expected to be generated during respiratory activities such as coughing, singing, or loud talking, additional studies examining the influence of particle size and regional deposition pattern within the respiratory tract on dose-response relationships and disease presentation are needed.

Finally, the present study utilized a viral isolate, hCoV-19/USA/WA-1/2020, from early in the pandemic that does not possess the mutations observed in more recent viral variants. Several of the more recent variants have been reported to be more transmissible [59] for reasons that remain unclear, but could potentially include increased viral shedding from infected individuals, greater infectivity in susceptible individuals, or enhanced environmental survival. Our laboratory recently demonstrated that the survival of an isolate of the B.1.1.7 lineage in aerosols was similar to that of earlier isolates across a range of environmental conditions, suggesting that enhanced environmental survival is likely not a factor in the increased transmissibility of this variant [19]. However, additional studies with the more recent variants are needed to evaluate whether they are more infectious and/or induce greater viral shedding than the variant utilized in the present study.

## Materials and methods

### Ethics statement

All research was conducted in compliance with the Animal Welfare Act and other federal statutes and regulations relating to animals and experiments involving animals and adheres to principles stated in the *Guide for the Care and Use of Laboratory Animals* [60], and approved by both the NBACC Institutional Animal Care and Use Committee and the DHS Compliance and Assurance Program Office. The facility where this research was conducted is fully accredited by the Association for Assessment and Accreditation of Laboratory Animal Care International.

### Virus and microtitration assay

SARS-CoV-2 hCoV-19/USA/WA-1/2020 (NR-53780, BEI Resources) was provided by NIAID and was stored at -80°C until use. The challenge stock was received directly from BEI Resources and was not passaged prior to inhalation exposures. This isolate was obtained from an oropharyngeal swab from a human case on January 19, 2020 and was deposited to BEI Resources by the Centers for Disease Control and Prevention. Sequence information for this isolate is available in GenBank under accession MT246667.1. Viral stocks were thawed and diluted, when appropriate, in culture media for growth (gMEM) consisting of Minimum Essential Medium (MEM; 11095–098, Gibco) with 10% heat-inactivated fetal bovine serum (FBS; 12107C, Atlanta Biologicals), 2 mM GlutaMAX (35-050-061, Life Technologies), 0.1 mM nonessential amino acid (NEAA; 11140–050, Life Technologies), 1 mM sodium pyruvate (11360–070, Life Technologies), and 1% antibiotic-antimycotic solution (15240–062, Life Technologies).

Vero cells (ATCC CCL-81) and Vero E6 cells (ATCC CRL-1586) were cultured at 37°C and 5% CO_2_ in gMEM and used for all infectivity assays and PRNT, respectively. A VIAFILL reagent dispenser (INTEGRA Biosciences Corp., Hudson, NH) was utilized to seed cells into 96-well, clear bottom plates for microtitration assays. Samples were serially diluted (10^0^ through 10^−4^) in plates containing 90–95% confluent monolayers of Vero cells. For each dilution, a total of 10 replicate wells were infected. Infected plates were then incubated at 37°C and 5% CO_2_ for 4 days followed by visual inspection of each well, using a Nikon TS100 microscope, for the presence of cytopathic effects (CPE). Media only negative controls were included on each plate. Viral titers in a sample, expressed as TCID_50_/mL of sample, were calculated according to the Spearman-Karber method.

### Animals

A total of 16 healthy, adult (4–8 years old) cynomolgus macaques (*Macaca fascicularis*; 8 male and 8 female), weighing 3–7 kilograms, were utilized in this study (Worldwide Primates Inc., Miami, FL and NIH Animal Center, Poolesville, MD). All animals were serologically naïve for SARS-CoV-2 prior to study as measured using the Euroimmun SARS-CoV-2 S1 ELSIA kit (El 2606–9601 G, Euroimmun Inc.).

### Inhalation exposures

Animals were anesthetized with Telazol (2–6 mg/kg i.m.) and placed in a supine position on a Lexan platform located within a Class III biosafety cabinet. Prior to exposure, respiratory parameters were measured using a calibrated pneumotach (25250 Accutach Flow Senor, BandB Medical Technologies, Carlsbad, CA. USA) attached to an anesthesia mask (Surgivet mask 32393B1, Patterson Veterinary) and a Hans Rudolph Smart Lab system (1140 Series Smart Lab, Hans Rudolph, Shawnee, KS. USA).

For inhalation exposures to SARS-CoV-2 aerosols, an anesthesia mask attached to an aerosol generation system was placed over the mouth and nares of the animal (Fig A in S1 Text). Initially, the system was supplied with clean, filtered air. Small particle aerosols were generated in the system using an air assist nozzle (IAZA5200415, The Lee Company), supplied with a liquid suspension of SARS-CoV-2 at 0.05 mL/minute and compressed air at 16 L/min. The generated aerosol was sampled continuously during exposure using two polytetrafluoroethylene (PTFE) filters (225–3708, SKC Inc.), each flowing at 5 L/min, and located upstream from the inhalation mask. Particle size was measured during aerosol generation using an Aerodynamic Particle Sizer (Model 3321, TSI Inc.) located upstream from the oronasal mask. The average temperature and relative humidity within the exposure system during aerosol generation were 26.9±0.9°C and 13.3±1.8%, respectively. Animals were allowed to breathe air containing the aerosolized virus until a target inhaled volume was reached, with an average exposure duration of 9.9±4.3 minutes (n = 16). At the end of the exposure period, aerosol generation was terminated and clean HEPA-filtered air was allowed to flow through the system for several minutes to allow residual aerosol to be purged from the system. Aerosol samplers continued to collect and the animal continued to breath from the system during this period.

Following removal of the animal from the exposure system, the PTFE filters were removed from the system, placed into 50 mL conical tubes containing 5 mL of gMEM, and vortexed to re-suspend collected virus. The re-suspended samples were assayed for infectivity using a viral microtitration assay, as already described. The infectious viral titer measured from the filters was used to estimate the infectious aerosol concentration for each test. The inhaled dose of infectious virus for each animal, in TCID_50_, was estimated as the product of the infectious aerosol concentration and the total volume of air inhaled during the exposure period. The deposited dose of infectious virus for each animal, in TCID_50_, was calculated as the product of the infectious aerosol concentration, the total volume of air inhaled during the exposure period, and the total deposition fraction, estimated using the rhesus macaque airway morphometry within the Multiple Path Particle Dosimetry Model (MPPD v.3.01; ARA Inc.) [61,62]. Rhesus macaque and cynomolgus macaque respiratory anatomy and ventilation parameters are similar for animals with similar body weights [63]. Therefore, the rhesus macaque deposition model is an adequate surrogate for the cynomolgus macaque.

### Post-exposure observations, sample collection, and analyses

Animals were observed twice daily for clinical signs of illness for 21 days post-exposure. Animals were housed in HEPA-filtered isolator cages (37324, Carter_2_ Systems, Inc.) during the pre- and post-exposure periods to minimize the potential for disease transmission among animals housed in the same room. The average (± standard deviation) relative humidity and temperature in the housing room were 50.4 ± 2.1% and 22.8 ± 1.4°C, respectively.

Body temperature was monitored continuously pre- and post-exposure utilizing implanted telemetry transmitters (L11-R or M00, Data Sciences International). Temperature monitoring began at least 3 days prior to exposure. Average temperatures were calculated every 15-minutes for the pre-exposure time period, and used to construct an average daily temperature profile for each animal. During the post-exposure period, fever was defined as an increase of 1°C above the time matched average from the pre-exposure period.

Animals were anesthetized on days 2, 4, 6, 8, 10, 14, and 21 post-exposure for blood/serum collection, nasopharyngeal and oropharyngeal swabs, and body weight measurement. Complete blood counts (CBCs) were determined on whole blood samples using a VetScan HM5 hematology analyzer (0023319, Abaxis) within 4 hours of collection. Coagulation parameters, specifically the partial thromboplastin time (PTT) and activated partial thromboplastin time (aPTT), were assessed on whole blood samples using a VetScan VSpro Coagulation analyzer (10023305, Abaxis) within 4 hours of collection.

Serum was utilized to assess serum chemistries, pro-inflammatory cytokine levels, and seroconversion. Serum chemistries were measured using a VetScan VS2 chemistry analyzer (1200–0000, Abaxis) with a Preventive Care Profile Plus VetScan Reagent Disc (500-047-12, Abaxis) within 4 hours of collection. Pro-inflammatory cytokine levels, specifically INF-γ, IL-1β, IL-2, IL-6, IL-8, and IL-10, were assessed using a V-Plex Proinflammatory Panel 1 (NHP) Kit (K15056G-2, Meso Scale Discovery) on a MESO QuickPlex SQ 120 (Meso Scale Discovery) per the manufacturer’s instructions for low volume samples. Seroconversion was monitored using the Euroimmun SARS-CoV-2 S1 ELSIA kit (El 2606–9601 G, Euroimmun Inc.) per manufacturer’s instructions. Absorbance for the ELISA assay was read on a SpectraMax iD5 Microplate Reader, which was used to determine sample positivity based on the kit instructions. Serum samples for cytokine and seroconversion analyses were batched and stored at -80°C until use.

For samples that were positive using the Euroimmun SARS-CoV-2 S1 ELISA kit, a PRNT was performed to estimate neutralizing titers. SARS-CoV-2 hCoV-19/USA/WA-1/2020 suspended in gMEM was diluted to yield 20–40 plaque-forming units per well. Two-fold serial dilutions of heat-inactivated serum in gMEM were mixed 1:1 with virus and incubated for 1 hour at 37°C and 5% CO_2_. Following removal of cell culture medium, 100 μL of the 1:1 inoculum was then added to the wells of a 6-well plate containing Vero E6 ATCC cell monolayers (95–100% confluence) and incubated for another hour at 37°C, with rocking at 15 minute intervals. Two mL of a primary agarose overlay, consisting of 1.0% SeaKem ME agarose (50010, Lonza) mixed 1:1 with complete 2X MEM (Temin’s modification, no phenol red; PN 11935046, Gibco), was added to each well and allowed to solidify at room temperature. Plates were returned to the 37°C incubator for 2 days prior to staining with a secondary agarose overlay containing 3% neutral red (N2889-100mL, Sigma-Aldrich). Plates were returned to the 37°C incubator, and the plaques were enumerated on a light box 24 ± 4 hours later. The titer required to reduce viral plaques by 50% (PRNT_50_) was estimated as the reciprocal of the highest dilution that resulted in at least a 50% reduction in the number of plaques relative to control wells.

Nasopharyngeal and oropharyngeal samples were collected using sterile specimen collection swabs (VF105-80, Vare Health), and immediately placed in a 15 mL conical tube containing 3 mL of gMEM. For nasopharyngeal swabs, both nares were swabbed using a single swab. Sample tubes were vortexed for 10 seconds to re-suspend collected material, and assayed for infectious virus by microtitration, as already described, or for the presence of viral RNA using reverse-transcription real-time PCR (RT-qPCR). For RT-qPCR, viral RNA was extracted from samples using the Qiagen Viral RNA Mini Kit per the manufacturer’s instructions. Quantification of SARS-CoV-2 RNA was accomplished using an Applied Biosystems 7500 Fast real-time PCR instrument and the SuperScript III One-Step RT-PCR MasterMix with Platinum Taq DNA Polymerase. Briefly, 15 μL of PCR master mix was combined with 5 μL of RNA per well. The master mix was comprised of sterile, molecular biology grade water, 1X SuperScript reaction mix, 1X SuperScript reverse transcriptase, 0.2 μM forward and reverse primers, and 0.1 μM FAM-labeled probe. The target of the PCR assay is a conserved region of the viral RNA-dependent RNA polymerase (RdRp) gene. The primer and probe sequences are based on sequences published by Corman *et al*. [64] but were modified to replace redundant bases to consensus bases. The sequences are as follows: Forward primer (5’- GTG AAA TGG TCA TGT GTG GCG G -3’), reverse primer (5’- CAA ATG TTA AAA ACA CTA TTA GCA TA-3’), FAM-labeled, double quencher probe (5’- /56-FAM/AGG TGG AAC /ZEN/CTC ATC AGG AGA TGC C/31ABkFQ/ -3’). Reaction plates also each contained a 7-point standard curve based on a synthetic RNA positive control representing the target amplicon (Bio-Synthesis) ranging from 10^0^ to 10^7^ RNA copies per 5 μL (2x10^2^ to 2x10^9^ RNA copies/mL) added to each well. Cycling conditions were run as follows: hold at 50°C for 30 minutes, 95°C for 10 minutes, followed by 40 cycles of 95°C for 15 seconds and 60°C for 1 minute. Quantification was determined by the number of cycles required to cross a threshold of 0.02 (C(t)). Viral RNA copies/mL of sample were interpolated from the standard curve for each plate. Samples below 2x10^4^ RNA copies/mL (10^2^ RNA copies/5 μL) were not utilized for quantification of RNA as the standards did not consistently amplify below this level.

### Data analysis

The dose response parameters for fever and seroconversion were estimated with a bivariate generalized linear model using the VGAM package in R [36]. Specifically, the marginal likelihood of each response was treated as a generalized linear model with a probit link, dependent upon the log_10_ transformed deposited dose values. The within subject dependency between seroconversion and fever was modeled with an odds ratio (R function VGAM::binom2.or) [65]. The overall quality of the bivariate model fit was evaluated via score test [66]. The strength of evidence in favor of treating the two endpoints with different dose response parameters was assessed via likelihood ratio test (LRT). Further, the strength of evidence in favor of the hypothesis that fever is dose dependent when conditioned on seroconversion was evaluated via LRT.

Confidence intervals and hypothesis test results that relied upon the bivariate dose response model were obtained via bootstrapping. In particular, the confidence intervals of the marginal median deposited doses for seroconversion and fever were assessed via the bias-corrected accelerated (BC_a_) bootstrap percentile interval method (B = 2000) [67]. The variance of the estimated difference in median deposited dose values was approximated via the delta method (R function emdbook::deltavar) [68]. This approximate variance was used to construct a standardized difference test statistic. The distribution of the standardized difference statistic was estimated from the bootstrap samples. This bootstrap distribution of the test statistic was leveraged to assess the significance of the difference between the median deposited dose values for seroconversion and fever.

As already noted, for comparison of viral shedding, cytokine levels, hematology, serum chemistry, and clotting parameters, animals were grouped by disease presentation, specifically those animals that had fever and seroconverted (n = 5), those that seroconverted but did not have fever (n = 5), and those animals that neither seroconverted nor had fever (n = 6). Analyses were performed using Prism (v. 8.4.3., GraphPad Software LLC.).

For viral shedding, the proportion of all post-exposure samples in each group that were positive for infectious virus and/or viral RNA were compared using Fisher’s exact tests. For cytokine levels (with the exception of IL-6), hematology, serum chemistry, and clotting parameters, post-exposure levels were compared to baseline levels within each group using repeated measures one-way ANOVA with a Holm-Sidak’s multiple comparisons test. For IL-6, baseline data were not normally distributed (P<0.0001 by D’Agostino and Pearson test). Therefore, post-exposure levels were compared to baseline levels within each group using the nonparametric Friedman test. Two points in the group of animals that did not seroconvert or develop fever were below the limit of detection of the assay. A value of zero was used for these two time points to allow correct ranking of the paired samples.

## Supporting information

S1 Text This file includes additional time course data for dosing, viral shedding, and clinical pathology referred to in the main text. (DOCX)
