# Supplementary material for: Seroconversion and fever are dose-dependent in a nonhuman primate model of inhalational COVID-19
Source: PLoS Pathog. 2021 Aug 23;17(8):e1009865. doi: 10.1371/journal.ppat.1009865 (PMC8412324; doi:10.1371/journal.ppat.1009865)
Supplement: S1 Text — (DOCX) [file ppat.1009865.s001.docx]

**Supplementary Data**


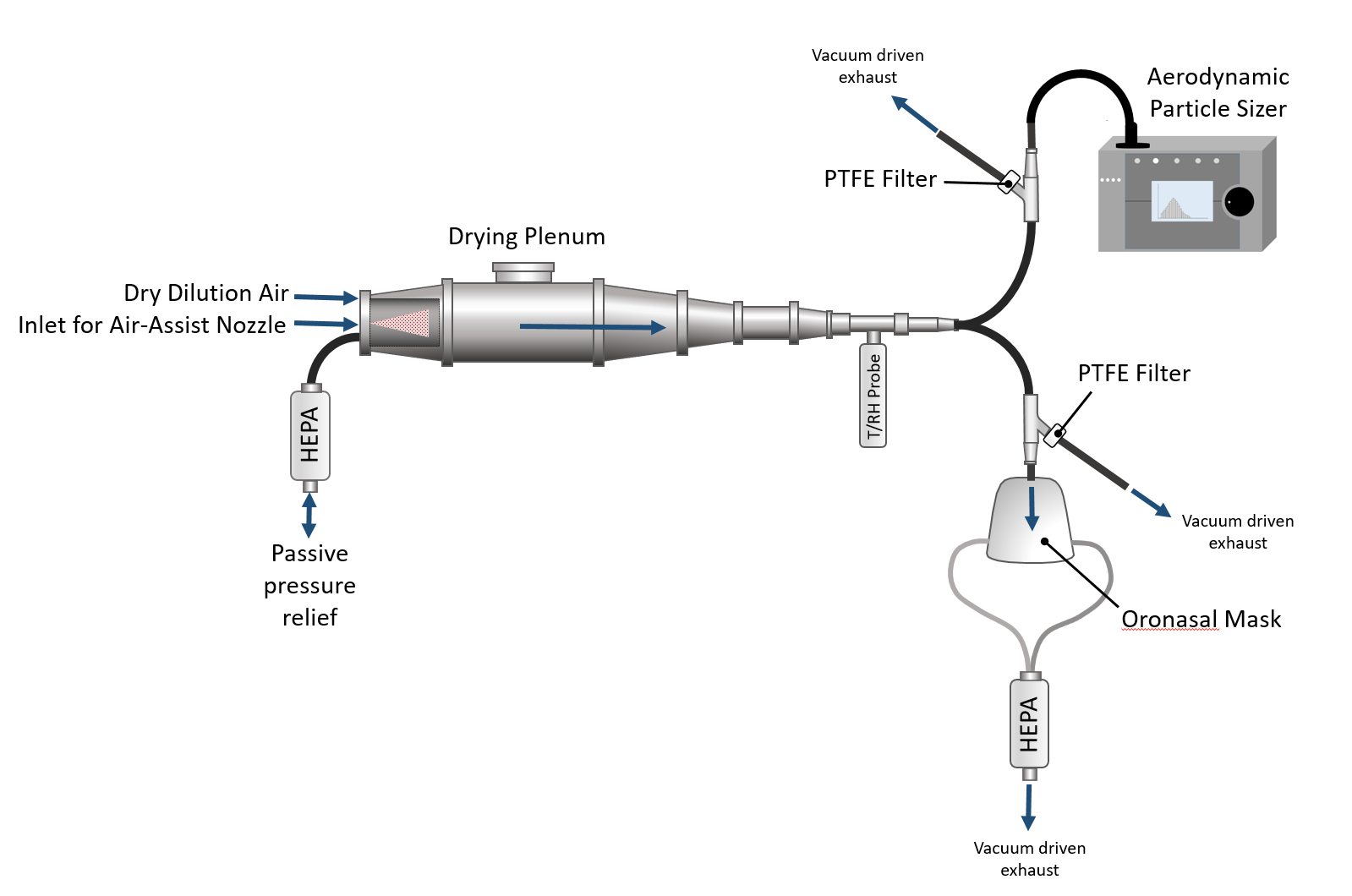


**Figure A – Inhalation Exposure System Schematic.** For inhalation exposures, a pediatric anesthesia mask attached to the aerosol generation system was placed over the mouth and nares of the animal. Small particle aerosols were generated using an air assist nozzle, supplied with a liquid suspension of SARS-CoV-2 at 0.0.05 mL/minute and compressed air at 16 L/min. The generated aerosols passed through a stainless steel plenum to allow evaporation and equilibration of the particles to occur. The equilibrated aerosol was sampled continuously during exposure using two PTFE filters, each flowing at 5 L/min, to estimate infectious aerosol concentration, and an Aerodynamic Particle Sizer, located just upstream from the inhalation mask, to measure the aerosol particle size distribution.

**Table A – Dose Summary for Individual Animals.** Inhaled and deposited doses are shown for each animal. Deposited dose was calculated as the product of the inhaled dose and the deposition fraction (f_dep_). Individual deposition fractions for each animal were estimated from the body weight, respiratory frequency (f) and tidal volume (TV), and the particle size distribution measured during exposure using the rhesus macaque airway morphometry within the Multiple Path Particle Dosimetry Model (version 3.01; ARA Inc.).

| **Subject** | **Gender** | **Age** (years) | **Inhaled Dose** | | **Deposited Dose** | | **f_dep_** | **TV**  (mL) | **f**  (min^-1^) | **Body Weight** (kg) | **MMAD** (µm) | **GSD** |
| --- | --- | --- | --- | --- | --- | --- | --- | --- | --- | --- | --- | --- |
|  |  |  | TCID_50_ | log_10_ TCID_50_ | TCID_50_ | log_10_ TCID_50_ |  |  |  |  |  |  |
| 1 | Female | 4.2 | 25 | 1.40 | 5 | 0.73 | 0.21 | 20 | 27 | 3.80 | 1.55 | 1.53 |
| 2 | Male | 5.9 | 43 | 1.63 | 7 | 0.82 | 0.16 | 27 | 31 | 6.34 | 1.50 | 1.62 |
| 3 | Male | 4.7 | 33 | 1.52 | 7 | 0.86 | 0.22 | 30 | 27 | 4.98 | 1.54 | 1.54 |
| 4 | Female | 7.4 | 65 | 1.82 | 17 | 1.23 | 0.26 | 24 | 37 | 3.42 | 1.50 | 1.57 |
| 5 | Female | 6.5 | 109 | 2.04 | 22 | 1.35 | 0.20 | 16 | 19 | 3.40 | 1.44 | 1.53 |
| 6 | Male | 4.7 | 336 | 2.53 | 58 | 1.76 | 0.17 | 20 | 18 | 5.18 | 1.48 | 1.54 |
| 7 | Female | 4.1 | 267 | 2.43 | 77 | 1.88 | 0.29 | 25 | 27 | 3.38 | 1.51 | 1.56 |
| 8 | Female | 7.0 | 393 | 2.59 | 78 | 1.89 | 0.20 | 16 | 20 | 3.48 | 1.47 | 1.54 |
| 9 | Male | 4.9 | 490 | 2.69 | 93 | 1.97 | 0.19 | 27 | 29 | 5.06 | 1.50 | 1.52 |
| 10 | Male | 4.7 | 585 | 2.77 | 96 | 1.98 | 0.16 | 16 | 16 | 4.46 | 1.46 | 1.54 |
| 11 | Male | 5.3 | 1443 | 3.16 | 206 | 2.31 | 0.14 | 21 | 29 | 5.32 | 1.48 | 1.52 |
| 12 | Female | 4.3 | 1198 | 3.08 | 366 | 2.56 | 0.31 | 28 | 31 | 3.34 | 1.50 | 1.53 |
| 13 | Male | 5.3 | 1618 | 3.21 | 582 | 2.77 | 0.36 | 54 | 15 | 5.44 | 1.42 | 1.61 |
| 14 | Female | 7.4 | 4945 | 3.69 | 825 | 2.92 | 0.17 | 28 | 30 | 4.18 | 1.28 | 1.52 |
| 15 | Female | 6.6 | 3656 | 3.56 | 904 | 2.96 | 0.25 | 25 | 26 | 3.28 | 1.24 | 1.49 |
| 16 | Male | 4.6 | 4350 | 3.64 | 906 | 2.96 | 0.21 | 22 | 21 | 5.30 | 1.26 | 1.48 |

**Table B – IgG ELISA Seroconversion Results.** Results (+/-) from the IgG ELISA are shown for each animal at each time point. Animals are arranged in order from lowest to highest deposited dose. Data on the presence of fever are also included for reference.

| **Subject** | **Disease Presentation** | | **Deposited Dose** | | **IgG ELISA Result (+/-)** | | | | | | | |
| --- | --- | --- | --- | --- | --- | --- | --- | --- | --- | --- | --- | --- |
|  |  |  |  |  | **Pre-exposure** | **Days Post Exposure** | | | | | | |
|  | **Sero-**  **conversion** | **Fever** | **TCID_50_** | **log_10_ TCID_50_** |  | **2** | **4** | **6** | **8** | **10** | **14** | **21** |
| 1 | - | - | 5 | 0.73 | - | - | - | - | - | - | - | - |
| 2 | - | - | 7 | 0.82 | - | - | - | - | - | - | - | - |
| 3 | - | - | 7 | 0.86 | - | - | - | - | - | - | - | - |
| 4 | - | - | 17 | 1.23 | - | - | - | - | - | - | - | - |
| 5 | - | - | 22 | 1.35 | - | - | - | - | - | - | - | - |
| 6 | + | - | 58 | 1.76 | - | - | - | - | - | - | + | + |
| 7 | - | - | 77 | 1.88 | - | - | - | - | - | - | - | - |
| 8 | + | - | 78 | 1.89 | - | - | - | - | - | - | + | + |
| 9 | + | - | 93 | 1.97 | - | - | - | - | - | - | - | + |
| 10 | + | + | 96 | 1.98 | - | - | - | - | - | - | + | + |
| 11 | + | - | 206 | 2.31 | - | - | - | - | - | - | + | + |
| 12 | + | - | 366 | 2.56 | - | - | - | - | - | - | - | + |
| 13 | + | + | 582 | 2.77 | - | - | - | - | - | - | + | + |
| 14 | + | + | 825 | 2.92 | - | - | - | - | - | - | - | + |
| 15 | + | + | 904 | 2.96 | - | - | - | - | - | + | + | + |
| 16 | + | + | 906 | 2.96 | - | - | - | - | - | - | + | + |

**Table C – Plaque Reduction Neutralization Test (PRNT_50_) Titers.** Results from the PRNT_50_ are shown for each animal at each time point. A value of <10 is considered negative. Greyed out cells indicate samples that were not assayed since the IgG ELISA was negative. Pre-exposure samples were only included as controls for animals that were assessed in the PRNT assay. Animals are arranged in order from lowest to highest deposited dose. Data on seroconversion and the presence of fever are also included for reference.

| **Subject** | **Disease Presentation** | | **Deposited Dose** | | **PRNT_50_ Titer** | | | | | | | |
| --- | --- | --- | --- | --- | --- | --- | --- | --- | --- | --- | --- | --- |
|  |  |  |  |  | **Pre-exposure** | **Days Post Exposure** | | | | | | |
|  | **Sero-**  **conversion** | **Fever** | **TCID_50_** | **log_10_ TCID_50_** |  | **2** | **4** | **6** | **8** | **10** | **14** | **21** |
| 1 | - | - | 5 | 0.73 |  |  |  |  |  |  |  |  |
| 2 | - | - | 7 | 0.82 |  |  |  |  |  |  |  |  |
| 3 | - | - | 7 | 0.86 |  |  |  |  |  |  |  |  |
| 4 | - | - | 17 | 1.23 |  |  |  |  |  |  |  |  |
| 5 | - | - | 22 | 1.35 | <10 |  |  |  |  |  |  | <10 |
| 6 | + | - | 58 | 1.76 | <10 |  |  |  |  | <10 | 80 | 160 |
| 7 | - | - | 77 | 1.88 |  |  |  |  |  |  |  |  |
| 8 | + | - | 78 | 1.89 | <10 |  |  |  |  | <10 | 160 | 640 |
| 9 | + | - | 93 | 1.97 | <10 |  |  |  |  | <10 | 80 | 80 |
| 10 | + | + | 96 | 1.98 | <10 |  |  |  |  | 320 | 640 | 640 |
| 11 | + | - | 206 | 2.31 | <10 |  |  |  |  | <10 | 160 | 160 |
| 12 | + | - | 366 | 2.56 | <10 |  |  |  |  | 160 | 640 | 640 |
| 13 | + | + | 582 | 2.77 | <10 |  |  |  |  | <10 | 160 | 320 |
| 14 | + | + | 825 | 2.92 | <10 |  |  |  |  | 20 | 160 | 80 |
| 15 | + | + | 904 | 2.96 | <10 |  |  |  |  | 80 | 320 | 640 |
| 16 | + | + | 906 | 2.96 | <10 |  |  |  |  | <10 | 40 | 160 |

**Table D – Viral Shedding in Oropharyngeal Swabs Measured by PCR.** PCR results for oropharyngeal swabs are shown for each animal at each time point. Animals are arranged in order from lowest to highest deposited dose. Data on seroconversion and the presence of fever are also included for reference; * denotes below limit of detection.

| **Subject** | **Disease Presentation** | | **Deposited Dose** | | **Viral Genomes (log_10_ RNA copies / mL)** | | | | | | | |
| --- | --- | --- | --- | --- | --- | --- | --- | --- | --- | --- | --- | --- |
|  |  |  |  |  | **Pre-exposure** | **Days Post Exposure** | | | | | | |
|  | **Sero-**  **conversion** | **Fever** | **TCID_50_** | **log_10_ TCID_50_** |  | **2** | **4** | **6** | **8** | **10** | **14** | **21** |
| 1 | - | - | 5 | 0.73 | * | * | * | * | * | * | * | * |
| 2 | - | - | 7 | 0.82 | * | * | * | * | * | * | * | * |
| 3 | - | - | 7 | 0.86 | * | * | * | * | * | * | * | * |
| 4 | - | - | 17 | 1.23 | * | * | * | * | * | * | * | * |
| 5 | - | - | 22 | 1.35 | * | * | * | 6.7 | * | * | * | * |
| 6 | + | - | 58 | 1.76 | * | 5.5 | 4.5 | * | * | * | * | * |
| 7 | - | - | 77 | 1.88 | * | 4.9 | * | * | * | * | * | * |
| 8 | + | - | 78 | 1.89 | * | 6.4 | * | 5.9 | * | * | * | * |
| 9 | + | - | 93 | 1.97 | * | * | * | * | * | * | * | * |
| 10 | + | + | 96 | 1.98 | * | 6.3 | * | * | 4.3 | * | * | * |
| 11 | + | - | 206 | 2.31 | * | 4.6 | * | * | * | * | * | * |
| 12 | + | - | 366 | 2.56 | * | * | * | * | * | * | * | * |
| 13 | + | + | 582 | 2.77 | * | 6.8 | * | 4.7 | * | * | * | * |
| 14 | + | + | 825 | 2.92 | * | * | * | 6.0 | 6.3 | * | * | * |
| 15 | + | + | 904 | 2.96 | * | * | * | * | * | * | * | * |
| 16 | + | + | 906 | 2.96 | * | 5.3 | * | * | 5.4 | * | * | * |

**Table E – Viral Shedding in Nasopharyngeal Swabs Measured by PCR.** PCR results for nasopharyngeal swabs are shown for each animal at each time point. Animals are arranged in order from lowest to highest deposited dose. Data on seroconversion and the presence of fever are also included for reference; * denotes below limit of detection.

| **Subject** | **Disease Presentation** | | **Deposited Dose** | | **Viral Genomes (log_10_ RNA copies / mL)** | | | | | | | |
| --- | --- | --- | --- | --- | --- | --- | --- | --- | --- | --- | --- | --- |
|  |  |  |  |  | **Pre-exposure** | **Days Post Exposure** | | | | | | |
|  | **Sero-**  **conversion** | **Fever** | **TCID_50_** | **log_10_ TCID_50_** |  | **2** | **4** | **6** | **8** | **10** | **14** | **21** |
| 1 | - | - | 5 | 0.73 | * | * | * | * | * | * | * | * |
| 2 | - | - | 7 | 0.82 | * | * | * | * | * | * | * | * |
| 3 | - | - | 7 | 0.86 | * | * | * | * | * | * | * | * |
| 4 | - | - | 17 | 1.23 | * | * | * | * | * | * | * | * |
| 5 | - | - | 22 | 1.35 | * | * | * | * | * | * | * | 5.4 |
| 6 | + | - | 58 | 1.76 | * | 4.8 | 8.5 | 7.6 | 5.7 | 6.7 | 4.6 | 4.6 |
| 7 | - | - | 77 | 1.88 | * | * | * | * | * | * | * | * |
| 8 | + | - | 78 | 1.89 | * | 5.0 | 6.1 | 8.1 | 6.4 | 4.8 | 4.5 | 4.4 |
| 9 | + | - | 93 | 1.97 | * | * | * | * | * | * | * | * |
| 10 | + | + | 96 | 1.98 | * | 5.6 | * | * | * | * | * | * |
| 11 | + | - | 206 | 2.31 | * | * | * | * | * | * | * | * |
| 12 | + | - | 366 | 2.56 | * | * | * | * | * | * | * | * |
| 13 | + | + | 582 | 2.77 | * | 4.8 | * | 5.0 | 5.7 | 6.1 | * | * |
| 14 | + | + | 825 | 2.92 | * | * | 5.3 | 8.2 | 6.3 | 4.7 | * | * |
| 15 | + | + | 904 | 2.96 | * | 4.6 | * | * | * | * | * | * |
| 16 | + | + | 906 | 2.96 | * | 8.7 | 4.8 | 6.2 | 7.1 | 5.6 | * | * |

**Table F – Viral Shedding in Oropharyngeal Swabs Measured by Microtitration.** Microtitration results for oropharyngeal swabs are shown for each animal at each time point. Animals are arranged in order from lowest to highest deposited dose. Data on seroconversion and the presence of fever are also included for reference; * denotes below limit of detection.

| **Subject** | **Disease Presentation** | | **Deposited Dose** | | **Viral Genomes (log_10_ RNA copies / mL)** | | | | | | | |
| --- | --- | --- | --- | --- | --- | --- | --- | --- | --- | --- | --- | --- |
|  |  |  |  |  | **Pre-exposure** | **Days Post Exposure** | | | | | | |
|  | **Sero-**  **conversion** | **Fever** | **TCID_50_** | **log_10_ TCID_50_** |  | **2** | **4** | **6** | **8** | **10** | **14** | **21** |
| 1 | - | - | 5 | 0.73 | * | * | * | * | * | * | * | * |
| 2 | - | - | 7 | 0.82 | * | * | * | * | * | * | * | * |
| 3 | - | - | 7 | 0.86 | * | * | * | * | * | * | * | * |
| 4 | - | - | 17 | 1.23 | * | * | * | * | * | * | * | * |
| 5 | - | - | 22 | 1.35 | * | * | * | 0.8 | * | * | * | * |
| 6 | + | - | 58 | 1.76 | * | * | * | * | * | * | * | * |
| 7 | - | - | 77 | 1.88 | * | * | * | * | * | * | * | * |
| 8 | + | - | 78 | 1.89 | * | 0.8 | * | * | * | * | * | * |
| 9 | + | - | 93 | 1.97 | * | * | * | * | * | * | * | * |
| 10 | + | + | 96 | 1.98 | * | 0.9 | * | * | * | * | * | * |
| 11 | + | - | 206 | 2.31 | * | * | * | * | * | * | * | * |
| 12 | + | - | 366 | 2.56 | * | * | * | * | * | * | * | * |
| 13 | + | + | 582 | 2.77 | * | 1.1 | * | * | * | * | * | * |
| 14 | + | + | 825 | 2.92 | * | * | * | * | 0.7 | * | * | * |
| 15 | + | + | 904 | 2.96 | * | * | * | * | * | * | * | * |
| 16 | + | + | 906 | 2.96 | * | 0.6 | * | * | * | * | * | * |

**Table G – Viral Shedding in Nasopharyngeal Swabs Measured by Microtitration.** Microtitration results for nasopharyngeal swabs are shown for each animal at each time point. Animals are arranged in order from lowest to highest deposited dose. Data on seroconversion and the presence of fever are also included for reference; * denotes below limit of detection.

| **Subject** | **Disease Presentation** | | **Deposited Dose** | | **Viral Genomes (log_10_ RNA copies / mL)** | | | | | | | |
| --- | --- | --- | --- | --- | --- | --- | --- | --- | --- | --- | --- | --- |
|  |  |  |  |  | **Pre-exposure** | **Days Post Exposure** | | | | | | |
|  | **Sero-**  **conversion** | **Fever** | **TCID_50_** | **log_10_ TCID_50_** |  | **2** | **4** | **6** | **8** | **10** | **14** | **21** |
| 1 | - | - | 5 | 0.73 | * | * | * | * | * | * | * | * |
| 2 | - | - | 7 | 0.82 | * | * | * | * | * | * | * | * |
| 3 | - | - | 7 | 0.86 | * | * | * | * | * | * | * | * |
| 4 | - | - | 17 | 1.23 | * | * | * | * | * | * | * | * |
| 5 | - | - | 22 | 1.35 | * | * | * | * | * | * | * | * |
| 6 | + | - | 58 | 1.76 | * | * | 3.2 | 0.6 | * | 0.8 | * | * |
| 7 | - | - | 77 | 1.88 | * | * | * | * | * | * | * | * |
| 8 | + | - | 78 | 1.89 | * | * | * | 1.0 | * | * | * | * |
| 9 | + | - | 93 | 1.97 | * | * | * | * | * | * | * | * |
| 10 | + | + | 96 | 1.98 | * | * | * | * | * | * | * | * |
| 11 | + | - | 206 | 2.31 | * | * | * | * | * | * | * | * |
| 12 | + | - | 366 | 2.56 | * | * | * | * | * | * | * | * |
| 13 | + | + | 582 | 2.77 | * | * | * | * | 0.6 | * | * | * |
| 14 | + | + | 825 | 2.92 | * | * | * | 2.8 | 1.0 | 0.6 | * | * |
| 15 | + | + | 904 | 2.96 | * | * | * | * | * | * | * | * |
| 16 | + | + | 906 | 2.96 | * | 3.3 | * | * | 0.9 | * | * | * |

**Table H – Serum IL-6 Concentrations.** Serum IL-6 concentrations are shown for each animal at each time point. Animals are arranged in order from lowest to highest deposited dose. Data on seroconversion and the presence of fever are also included for reference; * denotes below limit of detection.

| **Subject** | **Disease Presentation** | | **Deposited Dose** | | **IL-6 (pg/mL)** | | | | | | | |
| --- | --- | --- | --- | --- | --- | --- | --- | --- | --- | --- | --- | --- |
|  |  |  |  |  | **Pre-exposure** | **Days Post Exposure** | | | | | | |
|  | **Sero-**  **conversion** | **Fever** | **TCID_50_** | **log_10_ TCID_50_** |  | **2** | **4** | **6** | **8** | **10** | **14** | **21** |
| 1 | - | - | 5 | 0.73 | 5.7 | 4.7 | 7.7 | 4.3 | 4.7 | 10.9 | 4.0 | 2.5 |
| 2 | - | - | 7 | 0.82 | 78.0 | 41.4 | 54.4 | 30.1 | 67.1 | 38.3 | 23.7 | 21.2 |
| 3 | - | - | 7 | 0.86 | * | 1.1 | 1.8 | 3.1 | 1.6 | 2.8 | 1.6 | 1.3 |
| 4 | - | - | 17 | 1.23 | 0.7 | 6.8 | 1.9 | 2.1 | 1.0 | 1.8 | 2.2 | 4.5 |
| 5 | - | - | 22 | 1.35 | 0.3 | 0.7 | 1.1 | 1.5 | 2.8 | 0.7 | 0.5 | 1.4 |
| 6 | + | - | 58 | 1.76 | 11.6 | 17.3 | 15.7 | 16.2 | 14.2 | 10.7 | 11.0 | 12.1 |
| 7 | - | - | 77 | 1.88 | 0.6 | 0.5 | 0.6 | 1.3 | 0.5 | 0.7 | * | 0.5 |
| 8 | + | - | 78 | 1.89 | 0.6 | 4.5 | 3.8 | 1.2 | 2.4 | 1.6 | 1.4 | 3.1 |
| 9 | + | - | 93 | 1.97 | 2.8 | 4.3 | 1.8 | 2.0 | 3.2 | 6.1 | 3.7 | 2.1 |
| 10 | + | + | 96 | 1.98 | 1.0 | 6.0 | 6.8 | 1.4 | 2.0 | 0.8 | 0.9 | 1.6 |
| 11 | + | - | 206 | 2.31 | 2.9 | 12.7 | 3.2 | 3.8 | 1.8 | 1.1 | 1.5 | 1.4 |
| 12 | + | - | 366 | 2.56 | 0.7 | 1.3 | 1.3 | 1.5 | 1.4 | 0.9 | 0.4 | 0.5 |
| 13 | + | + | 582 | 2.77 | 0.4 | 2.7 | 1.9 | 1.1 | 1.9 | 1.6 | 1.0 | 0.8 |
| 14 | + | + | 825 | 2.92 | 4.1 | 5.4 | 3.4 | 5.9 | 6.8 | 7.1 | 0.8 | 1.5 |
| 15 | + | + | 904 | 2.96 | 2.4 | 38.7 | 6.1 | 3.6 | 3.5 | 1.5 | 2.7 | 2.6 |
| 16 | + | + | 906 | 2.96 | 0.7 | 100.5 | 6.0 | 2.8 | 1.4 | 2.0 | 1.5 | 2.7 |

**Table I – Serum IL-8 Concentrations.** Serum IL-6 concentrations are shown for each animal at each time point. Animals are arranged in order from lowest to highest deposited dose. Data on seroconversion and the presence of fever are also included for reference.

| **Subject** | **Disease Presentation** | | **Deposited Dose** | | **IL-8 (pg/mL)** | | | | | | | |
| --- | --- | --- | --- | --- | --- | --- | --- | --- | --- | --- | --- | --- |
|  |  |  |  |  | **Pre-exposure** | **Days Post Exposure** | | | | | | |
|  | **Sero-**  **conversion** | **Fever** | **TCID_50_** | **log_10_ TCID_50_** |  | **2** | **4** | **6** | **8** | **10** | **14** | **21** |
| 1 | - | - | 5 | 0.73 | 5.8 | 4.6 | 5.4 | 7.9 | 10.8 | 9.4 | 4.0 | 6.3 |
| 2 | - | - | 7 | 0.82 | 6.3 | 8.3 | 8.4 | 5.2 | 4.1 | 7.1 | 3.4 | 4.1 |
| 3 | - | - | 7 | 0.86 | 2.5 | 2.5 | 2.9 | 3.1 | 3.3 | 3.1 | 3.2 | 4.4 |
| 4 | - | - | 17 | 1.23 | 4.4 | 3.0 | 2.2 | 5.2 | 8.5 | 5.8 | 10.1 | 14.4 |
| 5 | - | - | 22 | 1.35 | 4.9 | 6.3 | 6.5 | 3.6 | 4.0 | 4.8 | 4.7 | 5.9 |
| 6 | + | - | 58 | 1.76 | 8.3 | 11.7 | 16.6 | 10.9 | 11.8 | 10.8 | 10.5 | 4.5 |
| 7 | - | - | 77 | 1.88 | 3.2 | 5.9 | 12.1 | 7.8 | 6.5 | 6.3 | 8.2 | 4.6 |
| 8 | + | - | 78 | 1.89 | 10.5 | 3.9 | 9.5 | 10.8 | 10.2 | 10.8 | 5.7 | 0.4 |
| 9 | + | - | 93 | 1.97 | 6.9 | 8.0 | 10.7 | 7.3 | 10.8 | 8.6 | 8.1 | 8.2 |
| 10 | + | + | 96 | 1.98 | 4.5 | 3.9 | 4.9 | 6.5 | 3.1 | 6.4 | 5.2 | 5.1 |
| 11 | + | - | 206 | 2.31 | 1.3 | 3.4 | 5.3 | 12.4 | 5.7 | 4.3 | 4.0 | 2.4 |
| 12 | + | - | 366 | 2.56 | 1.5 | 5.9 | 9.8 | 5.7 | 6.7 | 10.4 | 14.0 | 9.9 |
| 13 | + | + | 582 | 2.77 | 14.5 | 10.2 | 3.8 | 9.2 | 11.1 | 9.4 | 10.1 | 1.4 |
| 14 | + | + | 825 | 2.92 | 12.8 | 20.1 | 21.9 | 18.4 | 17.1 | 23.1 | 6.7 | 2.9 |
| 15 | + | + | 904 | 2.96 | 1.3 | 1.0 | 1.0 | 1.0 | 1.8 | 1.6 | 1.3 | 6.2 |
| 16 | + | + | 906 | 2.96 | 4.3 | 2.9 | 2.5 | 4.0 | 9.2 | 7.3 | 4.5 | 5.2 |

**Table J – Serum Neutrophil:Lymphocyte Ratio (NLR).** NLRs are shown for each animal at each time point. Animals are arranged in order from lowest to highest deposited dose. Data on seroconversion and the presence of fever are also included for reference.

| **Subject** | **Disease Presentation** | | **Deposited Dose** | | **NLR** | | | | | | | |
| --- | --- | --- | --- | --- | --- | --- | --- | --- | --- | --- | --- | --- |
|  |  |  |  |  | **Pre-exposure** | **Days Post Exposure** | | | | | | |
|  | **Sero-**  **conversion** | **Fever** | **TCID_50_** | **log_10_ TCID_50_** |  | **2** | **4** | **6** | **8** | **10** | **14** | **21** |
| 1 | - | - | 5 | 0.73 | 1.1 | 1.2 | 0.9 | 0.7 | 0.5 | 0.9 | 1.1 | 0.9 |
| 2 | - | - | 7 | 0.82 | 1.2 | 2.0 | 1.1 | 1.1 | 1.4 | 1.0 | 1.0 | 1.2 |
| 3 | - | - | 7 | 0.86 | 0.2 | 0.2 | 0.2 | 0.4 | 0.3 | 0.4 | 0.1 | 0.1 |
| 4 | - | - | 17 | 1.23 | 1.1 | 6.7 | 2.7 | 2.5 | 1.4 | 1.9 | 0.9 | 2.1 |
| 5 | - | - | 22 | 1.35 | 0.6 | 0.7 | 1.7 | 1.1 | 0.9 | 0.6 | 0.7 | 1.2 |
| 6 | + | - | 58 | 1.76 | 5.9 | 4.5 | 4.2 | 5.5 | 4.2 | 4.0 | 2.3 | 3.0 |
| 7 | - | - | 77 | 1.88 | 1.9 | 1.8 | 0.7 | 1.9 | 1.6 | 2.0 | 0.4 | 1.9 |
| 8 | + | - | 78 | 1.89 | 0.3 | 1.2 | 0.8 | 0.3 | 0.4 | 0.1 | 0.4 | 0.5 |
| 9 | + | - | 93 | 1.97 | 0.2 | 0.9 | 0.2 | 0.1 | 0.2 | 0.6 | 0.6 | 0.2 |
| 10 | + | + | 96 | 1.98 | 0.8 | 0.8 | 1.3 | 0.3 | 0.3 | 0.3 | 0.3 | 1.0 |
| 11 | + | - | 206 | 2.31 | 2.2 | 1.8 | 0.6 | 0.7 | 0.7 | 0.8 | 0.9 | 1.2 |
| 12 | + | - | 366 | 2.56 | 1.5 | 1.3 | 1.1 | 2.0 | 1.9 | 2.2 | 1.3 | 0.9 |
| 13 | + | + | 582 | 2.77 | 0.7 | 1.6 | 2.5 | 3.0 | 2.2 | 3.4 | 1.1 | 4.8 |
| 14 | + | + | 825 | 2.92 | 1.6 | 3.1 | 1.6 | 1.5 | 1.0 | 1.2 | 2.3 | 2.4 |
| 15 | + | + | 904 | 2.96 | 2.1 | 4.3 | 4.1 | 1.6 | 1.3 | 0.8 | 1.4 | 2.1 |
| 16 | + | + | 906 | 2.96 | 1.4 | 2.5 | 2.0 | 1.3 | 0.4 | 0.4 | 0.8 | 0.4 |

**Table K – Prothrombin Time (PT).** PTs are shown for each animal at each time point. Animals are arranged in order from lowest to highest deposited dose. Data on seroconversion and the presence of fever are also included for reference.

| **Subject** | **Disease Presentation** | | **Deposited Dose** | | **PT (seconds)** | | | | | | | |
| --- | --- | --- | --- | --- | --- | --- | --- | --- | --- | --- | --- | --- |
|  |  |  |  |  | **Pre-exposure** | **Days Post Exposure** | | | | | | |
|  | **Sero-**  **conversion** | **Fever** | **TCID_50_** | **log_10_ TCID_50_** |  | **2** | **4** | **6** | **8** | **10** | **14** | **21** |
| 1 | - | - | 5 | 0.73 | 20.1 | 20.1 | 19.8 | 20.2 | 21.0 | 19.4 | 20.0 | 19.9 |
| 2 | - | - | 7 | 0.82 | 20.7 | 19.3 | 20.2 | 19.8 | 19.1 | 19.0 | 19.5 | 19.8 |
| 3 | - | - | 7 | 0.86 | 20.5 | 20.4 | 20.0 | 20.1 | 20.6 | 19.1 | 20.2 | 20.9 |
| 4 | - | - | 17 | 1.23 | 20.4 | 20.6 | 19.3 | 19.5 | 19.8 | 19.6 | 19.2 | 19.3 |
| 5 | - | - | 22 | 1.35 | 20.1 | 20.7 | 20.1 | 19.9 | 20.5 | 18.9 | 19.8 | 20.1 |
| 6 | + | - | 58 | 1.76 | 21.8 | 20.4 | 20.1 | 20.4 | 20.7 | 19.4 | 19.5 | 19.7 |
| 7 | - | - | 77 | 1.88 | 19.3 | 19.1 | 19.0 | 19.6 | 18.6 | 19.0 | 19.5 | 19.2 |
| 8 | + | - | 78 | 1.89 | 20.2 | 20.0 | 19.8 | 20.0 | 19.6 | 18.6 | 19.6 | 19.8 |
| 9 | + | - | 93 | 1.97 | 20.2 | 20.4 | 20.0 | 19.7 | 20.3 | 19.3 | 19.3 | 21.2 |
| 10 | + | + | 96 | 1.98 | 21.1 | 21.3 | 20.7 | 19.7 | 19.3 | 19.9 | 21.1 | 20.2 |
| 11 | + | - | 206 | 2.31 | 20.0 | 19.8 | 20.9 | 19.2 | 19.8 | 19.2 | 20.4 | 20.1 |
| 12 | + | - | 366 | 2.56 | 19.0 | 18.6 | 20.2 | 18.8 | 18.9 | 19.0 | 20.4 | 19.4 |
| 13 | + | + | 582 | 2.77 | 21.4 | 21.6 | 22.5 | 20.5 | 21.3 | 20.8 | 21.3 | 20.6 |
| 14 | + | + | 825 | 2.92 | 19.8 | 19.3 | 19.5 | 18.7 | 19.4 | 18.3 | 18.9 | 19.6 |
| 15 | + | + | 904 | 2.96 | 19.4 | 19.2 | 18.4 | 18.4 | 19.1 | 18.6 | 19.2 | 18.6 |
| 16 | + | + | 906 | 2.96 | 20.8 | 20.6 | 20.7 | 19.7 | 20.2 | 19.6 | 19.9 | 21.0 |

**Table L – Activated Partial Thromboplastin Time (aPTT).** aPTTs are shown for each animal at each time point. Animals are arranged in order from lowest to highest deposited dose. Data on seroconversion and the presence of fever are also included for reference.

| **Subject** | **Disease Presentation** | | **Deposited Dose** | | **aPTT (seconds)** | | | | | | | |
| --- | --- | --- | --- | --- | --- | --- | --- | --- | --- | --- | --- | --- |
|  |  |  |  |  | **Pre-exposure** | **Days Post Exposure** | | | | | | |
|  | **Sero-**  **conversion** | **Fever** | **TCID_50_** | **log_10_ TCID_50_** |  | **2** | **4** | **6** | **8** | **10** | **14** | **21** |
| 1 | - | - | 5 | 0.73 | 90.7 | 91.6 | 87.4 | 89.9 | 89.1 | 85.7 | 94.7 | 96.4 |
| 2 | - | - | 7 | 0.82 | 78.7 | 80.9 | 74.8 | 80.3 | 75.6 | 76.0 | 83.1 | 82.4 |
| 3 | - | - | 7 | 0.86 | 84.1 | 86.6 | 82.5 | 84.0 | 82.9 | 87.3 | 87.2 | 89.5 |
| 4 | - | - | 17 | 1.23 | 87.1 | 84.1 | 85.7 | 86.4 | 87.0 | 85.6 | 85.9 | 86.1 |
| 5 | - | - | 22 | 1.35 | 84.7 | 86.1 | 82.1 | 84.3 | 84.0 | 82.0 | 86.3 | 87.3 |
| 6 | + | - | 58 | 1.76 | 89.1 | 84.9 | 86.2 | 86.9 | 87.4 | 90.5 | 86.6 | 74.4 |
| 7 | - | - | 77 | 1.88 | 90.1 | 83.1 | 86.5 | 86.0 | 95.3 | 86.7 | 88.6 | 90.3 |
| 8 | + | - | 78 | 1.89 | 85.8 | 87.5 | 95.8 | 90.5 | 87.3 | 91.1 | 96.0 | 87.9 |
| 9 | + | - | 93 | 1.97 | 87.9 | 90.5 | 95.6 | 86.9 | 93.2 | 89.4 | 87.7 | 86.5 |
| 10 | + | + | 96 | 1.98 | 87.1 | 89.4 | 91.4 | 93.1 | 85.2 | 89.5 | 94.6 | 88.6 |
| 11 | + | - | 206 | 2.31 | 90.4 | 86.3 | 89.9 | 98.0 | 92.7 | 94.1 | 91.1 | 92.5 |
| 12 | + | - | 366 | 2.56 | 80.1 | 92.3 | 96.7 | 86.7 | 86.3 | 91.8 | 92.7 | 88.1 |
| 13 | + | + | 582 | 2.77 | 97.7 | 87.7 | 91.5 | 92.7 | 92.2 | 92.7 | 100.6 | 93.3 |
| 14 | + | + | 825 | 2.92 | 78.4 | 88.9 | 93.1 | 87.3 | 89.2 | 87.3 | 89.9 | 87.4 |
| 15 | + | + | 904 | 2.96 | 87.9 | 87.5 | 91.9 | 88.6 | 90.0 | 82.1 | 87.9 | 90.7 |
| 16 | + | + | 906 | 2.96 | 87.7 | 91.7 | 102.5 | 94.9 | 98.2 | 93.0 | 90.4 | 90.8 |

**Table M – Blood Urea Nitrogen (BUN).** BUN levels are shown for each animal at each time point. Animals are arranged in order from lowest to highest deposited dose. Data on seroconversion and the presence of fever are also included for reference.

| **Subject** | **Disease Presentation** | | **Deposited Dose** | | **BUN (mg/dL)** | | | | | | | |
| --- | --- | --- | --- | --- | --- | --- | --- | --- | --- | --- | --- | --- |
|  |  |  |  |  | **Pre-exposure** | **Days Post Exposure** | | | | | | |
|  | **Sero-**  **conversion** | **Fever** | **TCID_50_** | **log_10_ TCID_50_** |  | **2** | **4** | **6** | **8** | **10** | **14** | **21** |
| 1 | - | - | 5 | 0.73 | 15.0 | 15.0 | 11.0 | 12.0 | 12.0 | 11.0 | 9.0 | 10.0 |
| 2 | - | - | 7 | 0.82 | 14.0 | 17.0 | 12.0 | 18.0 | 10.0 | 11.0 | 6.0 | 9.0 |
| 3 | - | - | 7 | 0.86 | 13.0 | 15.0 | 10.0 | 9.0 | 9.0 | 11.0 | 5.0 | 6.0 |
| 4 | - | - | 17 | 1.23 | 16.0 | 23.0 | 11.0 | 12.0 | 6.0 | 8.0 | 9.0 | 11.0 |
| 5 | - | - | 22 | 1.35 | 9.0 | 8.0 | 13.0 | 14.0 | 12.0 | 6.0 | 7.0 | 13.0 |
| 6 | + | - | 58 | 1.76 | 9.0 | 8.0 | 15.0 | 12.0 | 12.0 | 7.0 | 7.0 | 9.0 |
| 7 | - | - | 77 | 1.88 | 11.0 | 12.0 | 10.0 | 9.0 | 11.0 | 10.0 | 8.0 | 8.0 |
| 8 | + | - | 78 | 1.89 | 11.0 | 9.0 | 16.0 | 12.0 | 15.0 | 7.0 | 8.0 | 14.0 |
| 9 | + | - | 93 | 1.97 | 14.0 | 13.0 | 11.0 | 10.0 | 8.0 | 9.0 | 5.0 | 8.0 |
| 10 | + | + | 96 | 1.98 | 13.0 | 15.0 | 21.0 | 13.0 | 17.0 | 10.0 | 11.0 | 14.0 |
| 11 | + | - | 206 | 2.31 | 16.0 | 16.0 | 11.0 | 12.0 | 10.0 | 11.0 | 8.0 | 12.0 |
| 12 | + | - | 366 | 2.56 | 9.0 | 9.0 | 7.0 | 8.0 | 7.0 | 8.0 | 3.0 | 6.0 |
| 13 | + | + | 582 | 2.77 | 14.0 | 10.0 | 19.0 | 14.0 | 13.0 | 10.0 | 9.0 | 13.0 |
| 14 | + | + | 825 | 2.92 | 12.0 | 14.0 | 10.0 | 9.0 | 5.0 | 8.0 | 5.0 | 13.0 |
| 15 | + | + | 904 | 2.96 | 13.0 | 11.0 | 21.0 | 10.0 | 14.0 | 9.0 | 9.0 | 16.0 |
| 16 | + | + | 906 | 2.96 | 19.0 | 19.0 | 13.0 | 14.0 | 9.0 | 12.0 | 9.0 | 11.0 |

**Table N – Creatinine (CRE).** CRE levels are shown for each animal at each time point. Animals are arranged in order from lowest to highest deposited dose. Data on seroconversion and the presence of fever are also included for reference.

| **Subject** | **Disease Presentation** | | **Deposited Dose** | | **CRE (mg/dL)** | | | | | | | |
| --- | --- | --- | --- | --- | --- | --- | --- | --- | --- | --- | --- | --- |
|  |  |  |  |  | **Pre-exposure** | **Days Post Exposure** | | | | | | |
|  | **Sero-**  **conversion** | **Fever** | **TCID_50_** | **log_10_ TCID_50_** |  | **2** | **4** | **6** | **8** | **10** | **14** | **21** |
| 1 | - | - | 5 | 0.73 | 0.80 | 0.60 | 0.80 | 0.80 | 0.80 | 1.00 | 0.80 | 0.90 |
| 2 | - | - | 7 | 0.82 | 0.80 | 0.90 | 1.10 | 0.70 | 0.70 | 1.00 | 0.80 | 1.10 |
| 3 | - | - | 7 | 0.86 | 0.80 | 0.60 | 0.80 | 0.60 | 0.70 | 1.20 | 0.80 | 0.60 |
| 4 | - | - | 17 | 1.23 | 0.80 | 0.70 | 0.60 | 0.90 | 0.80 | 0.60 | 0.70 | 0.90 |
| 5 | - | - | 22 | 1.35 | 0.40 | 0.50 | 0.50 | 0.50 | 0.60 | 0.60 | 0.70 | 0.70 |
| 6 | + | - | 58 | 1.76 | 0.90 | 1.00 | 0.80 | 0.90 | 0.90 | 0.80 | 0.80 | 0.80 |
| 7 | - | - | 77 | 1.88 | 0.90 | 0.90 | 0.90 | 1.10 | 0.80 | 0.70 | 1.20 | 0.90 |
| 8 | + | - | 78 | 1.89 | 0.40 | 0.50 | 0.80 | 0.60 | 0.80 | 0.80 | 0.90 | 1.00 |
| 9 | + | - | 93 | 1.97 | 0.40 | 0.60 | 0.60 | 0.50 | 0.50 | 0.70 | 0.70 | 0.40 |
| 10 | + | + | 96 | 1.98 | 0.60 | 0.80 | 0.80 | 0.60 | 0.70 | 0.80 | 0.50 | 0.80 |
| 11 | + | - | 206 | 2.31 | 0.80 | 0.80 | 0.70 | 0.80 | 0.60 | 0.80 | 0.80 | 0.60 |
| 12 | + | - | 366 | 2.56 | 0.60 | 0.50 | 0.30 | 0.30 | 0.40 | 0.50 | 0.30 | 0.70 |
| 13 | + | + | 582 | 2.77 | 0.70 | 0.70 | 0.90 | 0.60 | 0.60 | 0.80 | 0.80 | 1.10 |
| 14 | + | + | 825 | 2.92 | 0.60 | 0.90 | 0.90 | 0.70 | 0.70 | 0.40 | 0.50 | 0.40 |
| 15 | + | + | 904 | 2.96 | 0.70 | 0.80 | 0.60 | 0.60 | 0.70 | 0.70 | 0.70 | 0.60 |
| 16 | + | + | 906 | 2.96 | 0.60 | 0.80 | 0.80 | 0.80 | 0.60 | 0.70 | 1.00 | 1.00 |

**Table O – BUN:CRE ratio.** BUN:CRE ratios are shown for each animal at each time point. Animals are arranged in order from lowest to highest deposited dose. Data on seroconversion and the presence of fever are also included for reference.

| **Subject** | **Disease Presentation** | | **Deposited Dose** | | **BUN:CRE** | | | | | | | |
| --- | --- | --- | --- | --- | --- | --- | --- | --- | --- | --- | --- | --- |
|  |  |  |  |  | **Pre-exposure** | **Days Post Exposure** | | | | | | |
|  | **Sero-**  **conversion** | **Fever** | **TCID_50_** | **log_10_ TCID_50_** |  | **2** | **4** | **6** | **8** | **10** | **14** | **21** |
| 1 | - | - | 5 | 0.73 | 18.75 | 25.00 | 13.75 | 15.00 | 15.00 | 11.00 | 11.25 | 11.11 |
| 2 | - | - | 7 | 0.82 | 17.50 | 18.89 | 10.91 | 25.71 | 14.29 | 11.00 | 7.50 | 8.18 |
| 3 | - | - | 7 | 0.86 | 16.25 | 25.00 | 12.50 | 15.00 | 12.86 | 9.17 | 6.25 | 10.00 |
| 4 | - | - | 17 | 1.23 | 20.00 | 32.86 | 18.33 | 13.33 | 7.50 | 13.33 | 12.86 | 12.22 |
| 5 | - | - | 22 | 1.35 | 22.50 | 16.00 | 26.00 | 28.00 | 20.00 | 10.00 | 10.00 | 18.57 |
| 6 | + | - | 58 | 1.76 | 10.00 | 8.00 | 18.75 | 13.33 | 13.33 | 8.75 | 8.75 | 11.25 |
| 7 | - | - | 77 | 1.88 | 12.22 | 13.33 | 11.11 | 8.18 | 13.75 | 14.29 | 6.67 | 8.89 |
| 8 | + | - | 78 | 1.89 | 27.50 | 18.00 | 20.00 | 20.00 | 18.75 | 8.75 | 8.89 | 14.00 |
| 9 | + | - | 93 | 1.97 | 35.00 | 21.67 | 18.33 | 20.00 | 16.00 | 12.86 | 7.14 | 20.00 |
| 10 | + | + | 96 | 1.98 | 21.67 | 18.75 | 26.25 | 21.67 | 24.29 | 12.50 | 22.00 | 17.50 |
| 11 | + | - | 206 | 2.31 | 20.00 | 20.00 | 15.71 | 15.00 | 16.67 | 13.75 | 10.00 | 20.00 |
| 12 | + | - | 366 | 2.56 | 15.00 | 18.00 | 23.33 | 26.67 | 17.50 | 16.00 | 10.00 | 8.57 |
| 13 | + | + | 582 | 2.77 | 20.00 | 14.29 | 21.11 | 23.33 | 21.67 | 12.50 | 11.25 | 11.82 |
| 14 | + | + | 825 | 2.92 | 20.00 | 15.56 | 11.11 | 12.86 | 7.14 | 20.00 | 10.00 | 32.50 |
| 15 | + | + | 904 | 2.96 | 18.57 | 13.75 | 35.00 | 16.67 | 20.00 | 12.86 | 12.86 | 26.67 |
| 16 | + | + | 906 | 2.96 | 31.67 | 23.75 | 16.25 | 17.50 | 15.00 | 17.14 | 9.00 | 11.00 |

**Table P – Body Weight.** Body weights are shown for each animal at each time point. Animals are arranged in order from lowest to highest deposited dose. Data on seroconversion and the presence of fever are also included for reference.

| **Subject** | **Disease Presentation** | | **Deposited Dose** | | **Body weight (kg)** | | | | | | | |
| --- | --- | --- | --- | --- | --- | --- | --- | --- | --- | --- | --- | --- |
|  |  |  |  |  | **Pre-exposure** | **Days Post Exposure** | | | | | | |
|  | **Sero-**  **conversion** | **Fever** | **TCID_50_** | **log_10_ TCID_50_** |  | **2** | **4** | **6** | **8** | **10** | **14** | **21** |
| 1 | - | - | 5 | 0.73 | 3.80 | 3.66 | 3.36 | 3.84 | 3.92 | 3.88 | 3.94 | 4.10 |
| 2 | - | - | 7 | 0.82 | 6.34 | 6.34 | 6.26 | 6.24 | 6.26 | 6.08 | 6.14 | 6.28 |
| 3 | - | - | 7 | 0.86 | 4.98 | 4.10 | 4.94 | 4.94 | 5.10 | 4.98 | 5.06 | 5.01 |
| 4 | - | - | 17 | 1.23 | 3.42 | 3.48 | 3.46 | 3.46 | 3.50 | 3.42 | 3.42 | 3.66 |
| 5 | - | - | 22 | 1.35 | 3.40 | 3.40 | 3.46 | 3.50 | 3.48 | 3.48 | 3.50 | 3.54 |
| 6 | + | - | 58 | 1.76 | 5.18 | 5.20 | 5.20 | 5.18 | 5.14 | 5.22 | 5.20 | 5.16 |
| 7 | - | - | 77 | 1.88 | 3.38 | 3.32 | 3.30 | 3.42 | 3.42 | 3.38 | 3.40 | 3.42 |
| 8 | + | - | 78 | 1.89 | 3.48 | 3.46 | 3.52 | 3.48 | 3.46 | 3.46 | 3.54 | 3.66 |
| 9 | + | - | 93 | 1.97 | 5.06 | 4.98 | 5.02 | 5.10 | 5.10 | 5.10 | 5.06 | 5.20 |
| 10 | + | + | 96 | 1.98 | 4.46 | 4.46 | 4.54 | 4.50 | 4.42 | 4.50 | * | 4.60 |
| 11 | + | - | 206 | 2.31 | 5.32 | 5.40 | 5.36 | 5.42 | 5.38 | 5.42 | 5.48 | 5.58 |
| 12 | + | - | 366 | 2.56 | 3.34 | 3.34 | 3.32 | 3.36 | 3.32 | 3.34 | 3.40 | 3.40 |
| 13 | + | + | 582 | 2.77 | 5.44 | 5.38 | 5.52 | 5.54 | 5.46 | 5.58 | 5.64 | 5.68 |
| 14 | + | + | 825 | 2.92 | 4.18 | 4.22 | 4.28 | 4.22 | 4.22 | 4.16 | 4.20 | 4.30 |
| 15 | + | + | 904 | 2.96 | 3.28 | 3.32 | 3.36 | 3.23 | 3.30 | 3.34 | 3.40 | 3.54 |
| 16 | + | + | 906 | 2.96 | 5.30 | 5.48 | 5.34 | 5.38 | 5.38 | 5.30 | 5.40 | 5.50 |

* denotes missing data point
